# Genetic or therapeutic neutralization of ALK1 reduces LDL transcytosis and atherosclerosis in mice

**DOI:** 10.1038/s44161-023-00266-2

**Published:** 2023-05-11

**Authors:** Sungwoon Lee, Hubertus Schleer, Hyojin Park, Erika Jang, Michael Boyer, Bo Tao, Ana Gamez-Mendez, Abhishek Singh, Ewa Folta-Stogniew, Xinbo Zhang, Lingfeng Qin, Xue Xiao, Lin Xu, Junhui Zhang, Xiaoyue Hu, Evanthia Pashos, George Tellides, Philip W. Shaul, Warren L. Lee, Carlos Fernandez-Hernando, Anne Eichmann, William C. Sessa

**Affiliations:** 1 Department of Pharmacology, Yale University School of Medicine, New Haven, CT USA; 2 Vascular Biology and Therapeutics Program, Yale University School of Medicine, New Haven, CT USA; 3 Genovac Antibody Discovery, Fargo, ND USA; 4 Department of Internal Medicine, Cardiovascular Research Center, Yale University School of Medicine, New Haven, CT USA; 5 Department of Cellular and Molecular Physiology, Yale University School of Medicine, New Haven, CT USA; 6 Department of Laboratory Medicine and Pathobiology, University of Toronto, Toronto, Ontario Canada; 7 Keenan Research Centre for Biomedical Science, St. Michael’s Hospital, Toronto, Ontario Canada; 8 W.M. Keck Biotechnology Resource Laboratory, Yale University School of Medicine, New Haven, CT USA; 9 Department of Comparative Medicine, Yale University School of Medicine, New Haven, CT USA; 10 Quantitative Biomedical Research Center, Department of Population & Data Sciences, University of Texas Southwestern Medical Center, Dallas, TX USA; 11 Department of Genetics, Yale University School of Medicine, New Haven, CT USA; 12 Yale Cardiovascular Research Center, Department of Internal Medicine, Yale University, School of Medicine, New Haven, CT USA; 13 Internal Medicine Research, Unit Pfizer Inc., Cambridge, MA USA; 14 Department of Surgery, Yale University School of Medicine, New Haven, CT USA; 15 Center for Pulmonary and Vascular Biology, Department of Pediatrics, University of Texas Southwestern Medical Center, Dallas, TX USA

**Keywords:** Cardiovascular diseases, Dyslipidaemias

## Abstract

Low-density lipoprotein (LDL) accumulation in the arterial wall contributes to atherosclerosis initiation and progression^1^. Activin A receptor-like type 1 (ACVRL1, called activin-like kinase receptor (ALK1)) is a recently identified receptor that mediates LDL entry and transcytosis in endothelial cells (ECs)^2,3^. However, the role of this pathway in vivo is not yet known. In the present study, we show that genetic deletion of ALK1 in arterial ECs of mice substantially limits LDL accumulation, macrophage infiltration and atherosclerosis without affecting cholesterol or triglyceride levels. Moreover, a selective monoclonal antibody binding ALK1 efficiently blocked LDL transcytosis, but not bone morphogenetic protein-9 (BMP9) signaling, dramatically reducing plaque formation in LDL receptor knockout mice fed a high-fat diet. Thus, our results demonstrate that blocking LDL transcytosis into the endothelium may be a promising therapeutic strategy that targets the initiating event of atherosclerotic cardiovascular disease.

## Abstract (web summary)

The bone morphogenetic protein (BMP) 9/10 receptor, ALK1, was recently shown to mediate LDL entry and transcytosis in endothelial cells. Here, Lee et al. show that genetic ablation of arterial endothelial ALK1 in mice limits atherosclerosis without affecting cholesterol or triglyceride levels and that a monoclonal antibody binding ALK1 efficiently blocks LDL transcytosis, but not BMP9 signaling, leading to a reduction in plaque burden.

## Main

Atherosclerotic cardiovascular disease (ASCD)^4^ including myocardial infarction (MI) and stroke is the leading cause of mortality worldwide. Atherosclerosis results in plaque accumulation, leading to arterial narrowing, plaque erosion or rupture triggering thrombosis and acute MI. Cholesterol-lowering drugs such as statins and PCSK9 inhibitors are highly effective in lowering plasma cholesterol levels; however, these drugs reduce relative cardiovascular risk by only approximately 35–40% (ref. ^5^).

LDL entry and retention in the vessel wall are central to the initiation of atherosclerosis^1,6,7^. Recent studies have shown that LDL entry into the endothelium occurs independent of the canonical LDL receptor (LDLR) and is mediated by ALK1 and/or scavenger receptor B1 (SR-B1)^2,3,8,9^. Both receptors are necessary for LDL transcytosis across the endothelium and, in the case of SR-B1, its loss reduces atherosclerosis in vivo^8^. However, the mechanism of LDL transcytosis in atherosclerosis remains incompletely understood.

In this Article, we provide evidence that ALK1 expression is associated with the development of coronary atherosclerosis in humans. We used genetic deletion of *Alk1* in arterial ECs (AECs) in mice to investigate ALK1 function in atherosclerosis. We then developed and validated a selective, anti-ALK1 antibody to examine the potential therapeutic effect in atherosclerosis initiation, progression and regression.

## Results

### ALK1 expression is higher in human atherosclerotic arteries and AEC-specific ALK1 deletion prevents atherosclerosis

**Fig. 1:**
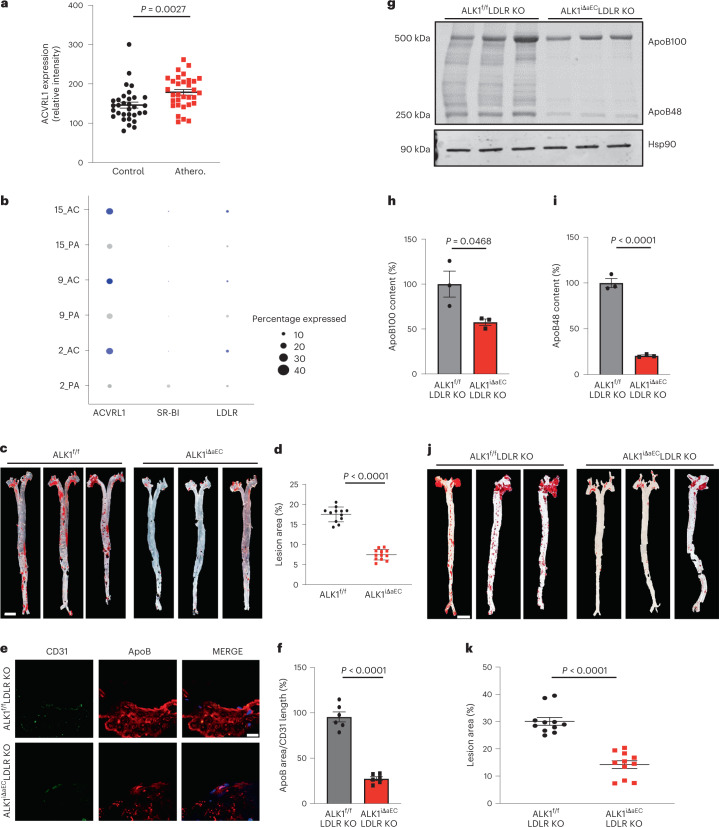
ALK1 expression is increased in human atherosclerotic arteries and ALK1 deletion prevents atherosclerosis via impaired LDL uptake. **a**, ALK1 expression in arteries from a cohort of control and atherosclerotic (Athero.) patients. Cohorts contained 32 subjects in each group. **b**, Expression profile showing relative expression of ACVRL1, SCARB1 and LDLR in AC and PA regions. For the largest cluster 2, *P* = 9.346198 × 10^−11^. **c**,**d**, Representative images (**c**) and analysis (**d**) of whole aorta showing accumulation of neutral lipids by ORO staining of *Alk1*^*f/f*^ and *Alk1*^*iΔaE*^ mice injected with mPCSK9 virus and fed a WD for 12 weeks (*n* = 12 mice per group). Scale bar, 2 mm. *P* < 0.0001 for **d**. Values show mean ± s.e.m. **e**,**f**, Representative immunostaining (**e**) and analysis (**f**) of endogenous apoB content per CD31 length/section in the lesser curvature of *Alk1*^*f/f*^*Ldlr*^−*/*−^ and *Alk1*^*iΔaEC*^*Ldlr*^−*/*−^ mice fed a WD for 4 weeks. Three images were counted per mouse in six mice per group. Scale bar, 20 μm. *P* < 0.0001 for **f**. Values show mean ± s.e.m. **g**–**i**, Western blotting (**g**) analysis of apoB100 (**h**) and apoB48 (**i**) proteins in whole aorta lysates from *Alk1*^*f/f*^*Ldlr*^−*/*−^ and *Alk1*^*iΔaEC*^*Ldlr*^−*/*−^ mice fed a WD for 4 weeks (*n* = 3 mice per group). *P* = 0.0468 for **h** and *P* < 0.0001 for **i**, respectively. Values show mean ± s.e.m. **j**,**k**, Representative images (**j**) and analysis (**k**) of whole aorta showing accumulation of neutral lipids by ORO staining of *Alk1*^*f/f*^;*Ldlr*^−*/*−^ and *Alk1*^*iΔaEC*^;*Ldlr*^−*/*−^ mice fed a WD for 12 weeks (*n* = 11 mice per group). Scale bar, 2 mm. *P* < 0.0001 for **k**. Values show mean ± s.e.m. All *P* values were calculated by two-tailed, unpaired Student’s *t*-test. Source data

**Extended Data Fig. 1:**
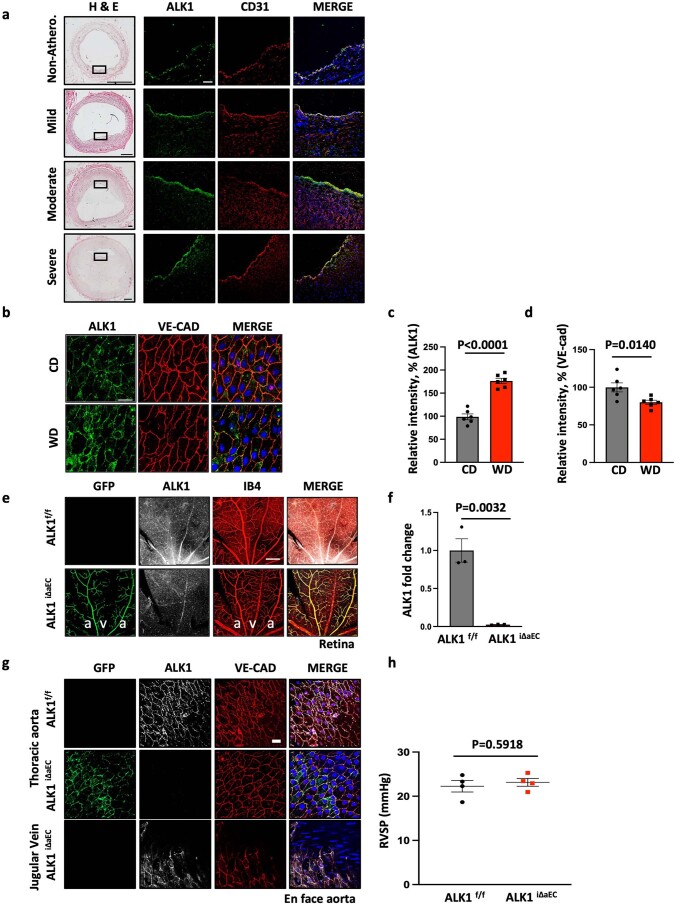
ALK1 expression is increased in atherosclerotic aorta ECs and BmxCre induces ALK1 deletion in arterial endothelial cells without changing right ventricle pressure. **a**, Representative images of human left main artery specimens sections stained for H&E (Scale bar: 600μm) and for ALK1 with CD31 (Scale bar: 20μm). Specimens collected from individual patient was categorized into Non-Athero, Mild, Moderate, and Severe depending on the severity (thickness) of plaques. **b-d**, Representative confocal images (**b**), and analysis of ALK1 (**c**) and VE-cad intensity (**d**). Two images were counted per mouse in three mice per group for a total of n = 6. Scale bar: 20μm. P < 0.0001 for c and P = 0.0140 for d. Values show mean ± S.E.M. *P* values were calculated by two-tailed unpaired t-test. Image J software was used to quantify the ALK1 and VE-cad intensity. **e**, Representative images of *Alk1*^*fl/fl*^*BmxCre*^*ERT2*^*mTmG* mouse adult retinal vessels, 4 months after tamoxifen injection. White: ALK1, green: GFP for mTmG, red: isolectin B4. Scale bar: 200μm. n = 3. **f**, Relative ALK1 expression from isolated aortic ECs of *Alk1*^*f/f*^ and *Alk1*^*iΔaE*^ mice, 4 months after tamoxifen injection. n = 5. P = 0.0032. Values show mean ± S.E.M. *P* values were calculated by two-tailed unpaired t-test. **g**, Representative confocal images of *Alk1*^*fl/fl*^*BmxCre*^*ERT2*^*mTmG* mouse thoracic aorta versus jugular vein vessels, 4 months after tamoxifen injection. White: ALK1, green:GFP for mTmG, red: VE-cadherin. Scale bar: 20μm. n = 3. Data are mean ± s.e.m. *P* values were calculated by two-tailed unpaired t-test. **h**, Right Ventricular Systolic Pressure (RVSP) of male *Alk1*^*f/f*^ and *Alk1*^*iΔaEC*^ mice at 6 weeks after tamoxifen injection, showing no difference between control and ALK1 deleted group. n = 4 male mice per group. P = 0.5918. Values show mean ± S.E.M. *P* values were calculated by two-tailed unpaired t-test. Source data

Previously, ALK1 staining was detected in the neointima, coronary endothelium, core of lesions and vicinity of lipids depending on the advancement of lesions^10^. To examine ALK1 expression in association with atherosclerotic lesions, we took advantage of two different publicly available databases. It is interesting that quantification of ALK1 messenger RNA levels shows higher levels of ALK1 in atherosclerotic versus normal arteries (Fig. 1a, microarray analysis from *n* = 32 patients for each cohort, and Fig. 1b, single-cell RNA-sequencing (scRNA-seq) analysis of EC populations from *n* = 3 patients for the atherosclerotic core (AC) and proximal adjacent (PA) groups). Increased ALK1 protein levels were also observed in both human specimens and mice lacking the LDLR (*Ldlr*^−*/*−^) fed a western diet (WD) for 6 weeks (Extended Data Fig. 1a–d). However, to assess the causal role of ALK1 in vivo is challenging because global or postnatal deletion of ALK1 in ECs results in lethality^2,11,12^. To overcome this challenge, we crossed *Alk1*^*f/f*^ mice on an mTmG reporter background to mice expressing the inducible estrogen receptor (ERT2) Cre recombinase under control of the bone marrow x (Bmx) promoter (Bmx-Cre-ERT2). The Bmx promoter enables Cre recombinase activity in AECs^13^ and does not promote arterial–venous malformations as is seen in *Alk1*^*f/f*^ mice crossed with other EC-selective promoters^14^. AEC-specific deletion of ALK1 was confirmed in adult mouse retina (green fluorescent protein (GFP)-positive arteries depleted of ALK1) and in en face images of ECs of aorta (GFP positive depleted of ALK1) versus jugular vein (GFP negative that are ALK1 positive) and in isolated aorta ECs (mRNA). Most importantly, the mice are viable and were used for long-term atherosclerosis studies (*Alk1*^*iΔaEC*^; Extended Data Fig. 1e–g). Given the important role of BMP signaling in the pulmonary circulation^15–17^, we assessed pulmonary pressures. AEC-specific deletion of ALK1 did not affect right ventricular systolic pressures (RVSPs) measured in adult *Alk1*^*iΔaEC*^ mice compared with control mice (Extended Data Fig. 1h).

**Extended Data Fig. 2:**
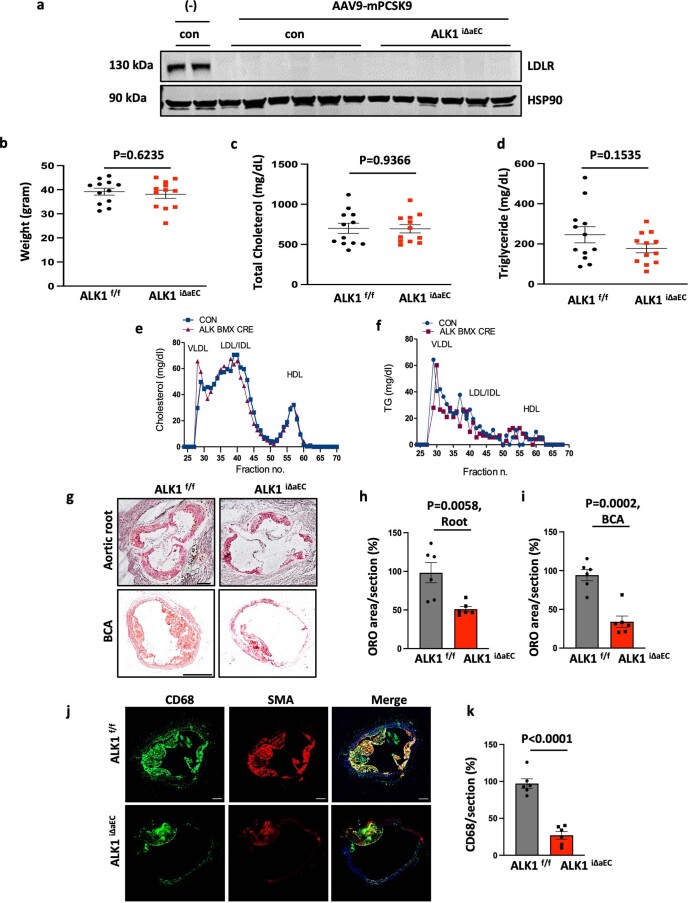
Arterial EC ALK1 deficiency reduces the atherosclerosis progression. **a**, LDL receptor protein levels from livers of *Alk1*^*f/f*^ and *Alk1*^*iΔaEC*^ mice received AAV9-mPCSK9 and fed a Western diet for 12 weeks. n = 6 mice per group. **b-d**, Analysis of weight (**b**), plasma total cholesterol (**c**) and triglycerides (**d**) levels from *Alk1*^*f/f*^ and *Alk1*^*iΔaEC*^ mice received AAV9-mPCSK9 through retro-orbital sinus delivery and fed a Western diet for 12 weeks. n = 12 mice per group. P = 0.6235 for b, P = 0.9366 for c, and P = 0.1535 for d. Values show mean ± S.E.M. *P* values were calculated by two-tailed unpaired t-test. **e**,**f**, Lipoprotein profiles of plasma total cholesterol (**e**) and triglyceride (**f**) from *Alk1*^*f/f*^ and *Alk1*^*iΔaEC*^ mice received AAV9-mPCSK9 and fed a Western diet for 12 weeks. n = 6 mice per group. **g-i**, Representative ORO-stained images (**g**), and quantification of neutral lipid content of aortic roots (**h**) and brachiocephalic arteries (**i**). Scale bars: 500μm and 100μm, for aortic root and brachiocephalic arteries, respectively. n = 6. P = 0.0058 for h and P = 0.0002 for i. Values show mean ± S.E.M. *P* values were calculated by two-tailed unpaired t-test. **j,k**, Confocal images (**j**) and analysis (**k**) of brachiocephalic arterial sections for macrophage infiltration, green: CD68, red: SMA, blue: nuclei. Scale bar: 100μm. P < 0.0001 for k. Values show mean ± S.E.M. *P* values were calculated by two-tailed unpaired t-test. All sectioned samples are prepared from *Alk1*^*f/f*^ and *Alk1*^*iΔaEC*^ mice received AAV9-mPCSK9 and fed a Western diet for 12 weeks. n = 6. All aortic root and brachiocephalic arteries were sectioned at 6μm. Image J software was used to quantify the samples. Values show mean ± S.E.M. *P* values were calculated by two-tailed unpaired t-test. Source data

To study whether arterial ALK1 affects atherosclerosis in *Alk1*^*iΔaEC*^ mice, AAV9-mPCSK9 was injected to delete LDLR followed by feeding a WD for 12 weeks (see deletion of hepatic LDLR, Extended Data Fig. 2a). No substantial changes in body weight, circulating lipids or lipoprotein profiles (Extended Data Fig. 2b–f) were observed, although a remarkable reduction in neutral lipid content was observed in the aorta of *Alk1*^*iΔaEC*^ mice as assessed by Oil Red O (ORO) staining (Fig. 1c,d). Similar changes were observed in both aortic roots and the brachiocephalic artery (BCA) sections (Extended Data Fig. 2g–i), as well as a reduction in macrophage infiltration assessed by CD68 staining (Extended Data Fig. 2j,k).

**Extended Data Fig. 3:**
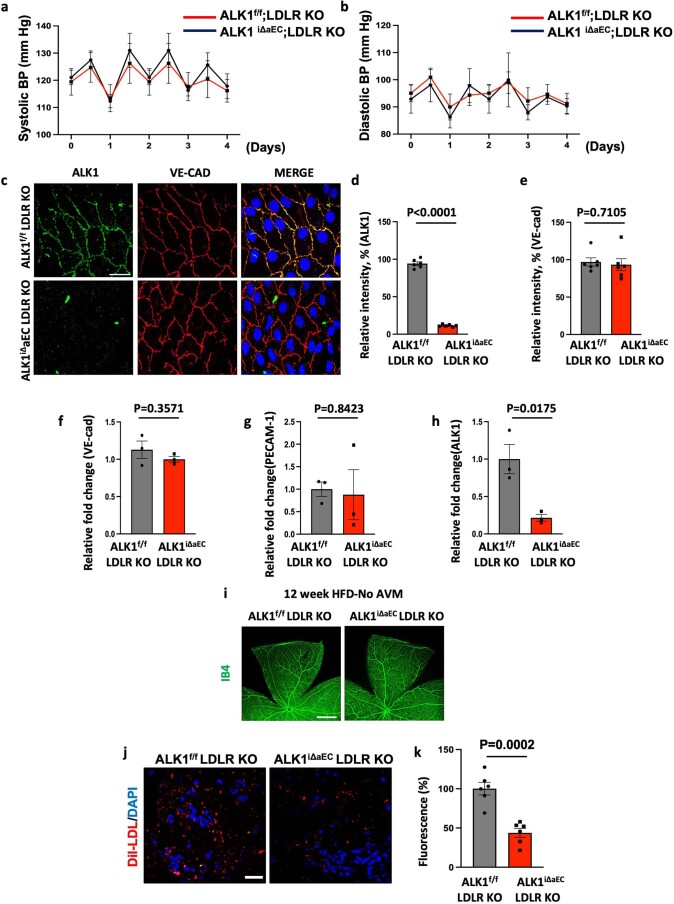
3 Arterial EC specific ALK1 deletion does not affect blood pressure or vascular tone and reduces DiI-LDL uptake. **a,b**, Telemetric systolic (**a**) and diastolic (**b**) BP of *Alk1*^*f/f*^*Ldlr*^−*/*−^ and *Alk1*^*iΔaEC*^*Ldlr*^−*/*−^ mice monitored over 4 consecutive days at 12 weeks after tamoxifen injection, showing no difference between control and ALK1 deleted group. n = 5 male mice per group. **c-e**, Representative confocal images (**c**), and analysis of ALK1 (**d**) and VE-cad intensity (**e**). Two images were counted per mouse in three mice per group for a total of n = 6. Scale bar: 20μm. P < 0.0001 for d and P = 0.7105 for e. Values show mean ± S.E.M. Image J software was used to quantify the ALK1 and VE-cad intensity. **f-h**, Relative VE-cad (**f**), PECAM-1 (**g**), and ALK1 (**h**) expression from isolated aortic ECs of *Alk1*^*f/f*^*Ldlr*^−*/*−^ and *Alk1*^*iΔaEC*^*Ldlr*^−*/*−^ mice fed a Western diet for 6 weeks. n = 3. P = 0.3571 for f, P = 0.8423 for g, and P = 0.0175 for h. Values show mean ± S.E.M. *P* values were calculated by two-tailed unpaired t-test. **i**, Isolectin B4 staining of retinal vessels of *Alk1*^*f/f*^*Ldlr*^−*/*−^ and *Alk1*^*iΔaEC*^*Ldlr*^−*/*−^ mice fed a Western diet for 12 weeks. n = 6. Scale bar: 400μm. **j,k**, Representative en face immunofluorescence (**j**) analysis (**k**) of injected DiI-LDL in the ascending aorta of *Alk1*^*f/f*^ *Ldlr*^−*/*−^ and *Alk1*^*iΔaEC*^ *Ldlr*^−*/*−^ mice. Three images were counted per mouse in two mice per group for a total of n = 6. Scale bar: 20μm. P = 0.0002 for k. Values show mean ± S.E.M. All *P* values were calculated by two-tailed unpaired t-test. Source data

**Extended Data Fig. 4:**
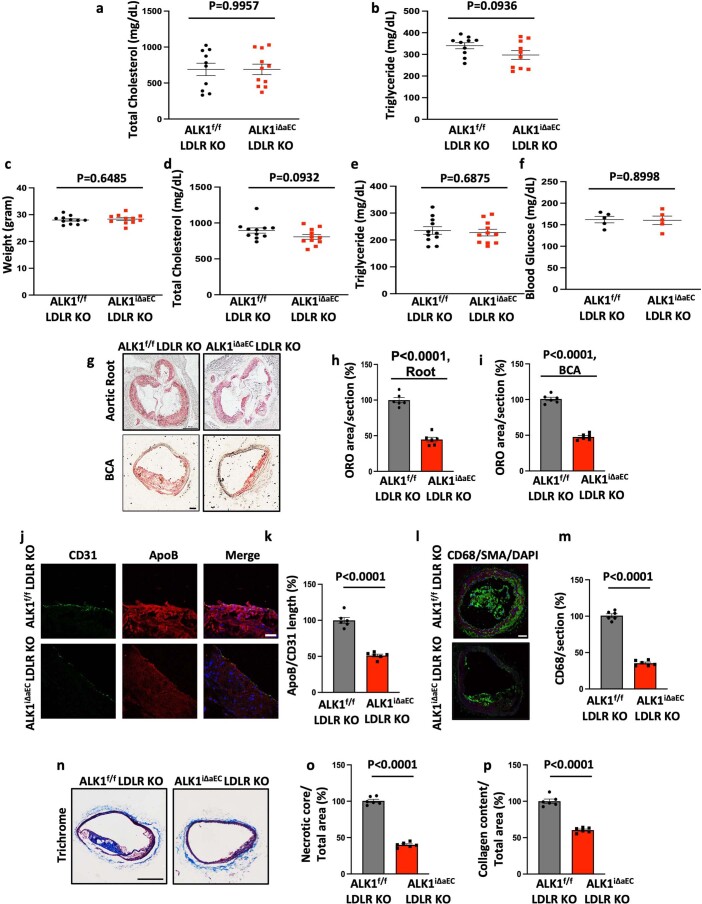
Arterial EC ALK1 deficiency reduces the progression of atherosclerosis without changing lipid metabolism. **a,b**, Plasma total cholesterol (**a**) and triglyceride (**b**) level from *Alk1*^*f/f*^ *Ldlr*^−*/*−^ and *Alk1*^*iΔaEC*^ *Ldlr*^−*/*−^ mice fed a Western diet for 4 weeks. n = 10. P = 0.9957 for a and P = 0.0936 for b. Values show mean ± S.E.M. **c-f**, Analysis of weight (**c**), plasma total cholesterol (**d**), triglycerides (**e**), and blood glucose (**f**) levels from *Alk1*^*f/f*^ *Ldlr*^−*/*−^ and *Alk1*^*iΔaEC*^ *Ldlr*^−*/*−^ mice fed a Western diet for 12 weeks. n = 10 mice per group. P = 0.6485 for c, P = 0.0932 for d, P = 0.6875 for e, and P = 0.8998 for f. Values show mean ± S.E.M. **g-i**, Representative ORO-stained images (**g**), and quantification of neutral lipid content of aortic roots (**h**) and brachiocephalic arteries (**i**). Scale bars: 500μm and 100μm, for aortic root and brachiocephalic arteries, respectively. P < 0.0001 for h and P < 0.0001 for i. Values show mean ± S.E.M. **j,k**, Confocal images (**j**) and analysis (**k**) of brachiocephalic artery sections for apoB content, red: apoB, green: CD31, blue: nuclei. Scale bar: 20μm. P < 0.0001 for k. Values show mean ± S.E.M. **l,m**, Confocal images (**l**) and analysis (**m**) of brachiocephalic arterial sections for macrophage infiltration, green: CD68, blue: nuclei. Scale bar: 100μm. P < 0.0001 for m. Values show mean ± S.E.M. **n-p**, Trichrome staining (**n**) and analysis of brachiocephalic artery sections for plaque necrosis (**o**) and collagen content (**p**). Scale bar: 100μm. P < 0.0001 for both o and p. Values show mean ± S.E.M. All samples are prepared from *Alk1*^*f/f*^*Ldlr*^−*/*−^ and *Alk1*^*iΔaEC*^*Ldlr*^−*/*−^ mice fed a Western diet for 12 weeks. All aortic root and brachiocephalic arteries were sectioned at 6μm. n = 7 mice per group. Image J software was used to quantify the samples. All *P* values were calculated by two-tailed unpaired t-test. Source data

To generate a genetically modified model of atherosclerosis, we bred mice lacking ALK1 specifically in arterial endothelium to *Ldlr*^−*/*−^ mice. To assess overall hemodynamics, blood pressure telemetry in conscious mice was performed. AEC deletion of ALK1 did not change systolic or diastolic pressures in *Alk1*^*iΔaEC*^;*Ldlr*^−*/*−^ mice compared with control mice (Extended Data Fig. 3a,b). Complete deletion of ALK1 was confirmed in en face aortic images of *Alk1*^*iΔaEC*^;*Ldlr*^−*/*−^ mice fed a WD for 6 weeks, without changing VE-cadherin (VE-cad) levels (Extended Data Fig. 3c–e). ALK1 mRNA level was also deleted in the AECs of *Alk1*^*iΔaEC*^;*Ldlr*^−*/*−^ mice fed a WD for 6 weeks, without changing VE-cad or platelet endothelial cell adhesion molecule 1 (PECAM-1) levels (Extended Data Fig. 3f–h). Furthermore, no vascular malformations were observed in retinal vessels of *Alk1*^*iΔaEC*^;*Ldlr*^−*/*−^ mice fed a WD for 12 weeks (Extended Data Fig. 3i). Finally, we assessed the uptake of LDL into the vessel wall. The uptake of injected DiI-labeled human native LDL (DiI-nLDL) into ECs was substantially reduced in the aorta of *Alk1*^*iΔaEC*^;*Ldlr*^−*/*−^ mice compared with *Alk1*^*f/f*^;*Ldlr*^−*/*−^ controls (Extended Data Fig. 3j,k). As atherogenic particles such as very-low-density lipoprotein, intermediate-density lipoprotein^2^, LDL and lipoprotein(a) contain one molecule of apolipoprotein B (apoB)100 per particle, quantification of apoB100 is a direct measurement of the number of atherogenic particles^5,18^. Thus, we assessed the apoB content of the aorta by confocal imaging of apoB in the lesser curvature region of the aortic arch and by western blotting of whole aortic lysates after 4 weeks of a WD. The loss of ALK1 in ECs did not impact circulating lipids (Extended Data Fig. 4a,b), but dramatically reduced apoB content (Fig. 1e,f) compared with *Alk1*^*f/f*^;*Ldlr*^−*/*−^ controls. In addition, western blotting with an antibody that recognizes the common amino terminus of apoB100 and apoB48 shows a marked reduction in the content of the lipoproteins in aortic lysates from *Alk1*^*iΔaEC*^;*Ldlr*^−*/*−^ mice (Fig. 1g–i).

To further examine the impact of ALK1 deletion on the progression of atherosclerosis, mice were fed a WD for 12 weeks followed by analysis of lipid parameters and plaque formation. Arterial endothelial ALK1 deletion in *Alk1*^*iΔaEC*^;*Ldlr*^−*/*−^ mice did not alter weight, total cholesterol, triglyceride or blood glucose levels (Extended Data Fig. 4c–f), but markedly reduced plaque formation (Fig. 1j,k). Next, we characterized aspects of lesion composition by quantifying lipid deposition, inflammation and collagen content in aortic roots and the BCA. There was a substantial reduction in lipid accumulation (Extended Data Fig. 4g–i) in both vessel segments and a marked decrease in the apoB content of the BCA of *Alk1*^*iΔaEC*^;*Ldlr*^−*/*−^ compared with *Alk1*^*f/f*^;*Ldlr*^−*/*−^ controls (Extended Data Fig. 4j,k). Analysis of CD68^+^ macrophage content revealed marked reduction in macrophage infiltration (Extended Data Fig. 4l,m) and less plaque necrosis and collagen content (Extended Data Fig. 4n–p) consistent with smaller lesions in *Alk1*^*iΔaEC*^;*Ldlr*^−*/*−^ mice. Complete blood counts did not differ between the two strains (Supplementary Table 1). Collectively, these results show that arterial endothelial ALK1 plays an important role in the development of atherosclerosis by regulating the uptake of circulating apoB-containing lipoproteins into the vessel wall without impacting plasma lipid metabolism, circulating cells or blood pressure.

### Anti-ALK1 antibody blocks LDL trafficking but not BMP9 signaling

**Fig. 2:**
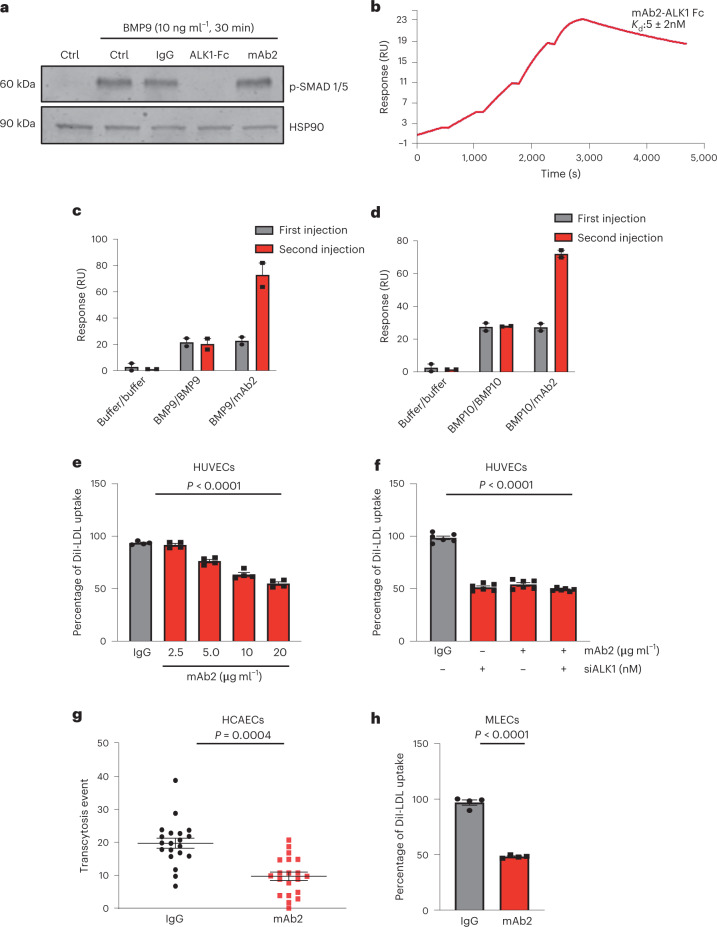
A selective, anti-ALK1 monoclonal antibody (mAb2) binds ALK1 and blocks LDL uptake and transcytosis but not BMP9 signaling. **a**, Western blot showing mAb2 (20 μg ml^−1^) not blocking BMP9 (10 ng ml^−1^)-mediated SMAD 1/5 phosphorylation. The negative control is nonimmune IgG (20 μg ml^−1^) and the positive control to quench BMP9 signaling is ALK1-Fc (1 μg ml^−1^). The experiment was repeated three times. Ctrl, control. **b**, SPR analysis of binding of mAb2 to ALK1-Fc. RU, relative units. **c**,**d**, SPR analysis of binding of mAb2 to ALK1-Fc saturated with BMP9 (**c**) or BMP10 (**d**). Two consecutive injections were delivered to the Fc-ALK1 surface (*n* = 2). **e**, Analysis of DiI-LDL uptake into HUVECs incubated with increasing concentrations of IgG or mAb2 (*n* = 3). *P* < 0.0001. Values show mean ± s.e.m. **f**, Comparison of DiI-LDL uptake into either HUVECs treated with mAb2 or ALK1-silenced HUVECs treated with mAb2 (20 μg ml^−1^) (*n* = 6). *P* < 0.0001. Values show mean ± s.e.m. **g**, Relative DiI-LDL transcytosis events measured by TIRF microscopy between IgG- and mAb2-treated HCAECs. IgG or mAb2 was used at 20 μg ml^−1^. The experiment was repeated twice and the representative dots were combined from the two independent experiments. *P* = 0.0004. Values show mean ± s.e.m. **h**, DiI-LDL uptake into *Ldlr*^−*/*−^ MLECs treated with the mAb2. IgG or mAb2 used at 20 μg ml^−1^ (*n* = 4). *P* < 0.0001. Values show mean ± s.e.m. *P* values were calculated by one-way ANOVA with Sidak’s multiple comparisons test for **e** and **f** and by two-tailed, unpaired Student’s *t*-test for **g** and **h**. Source data

**Extended Data Fig. 5:**
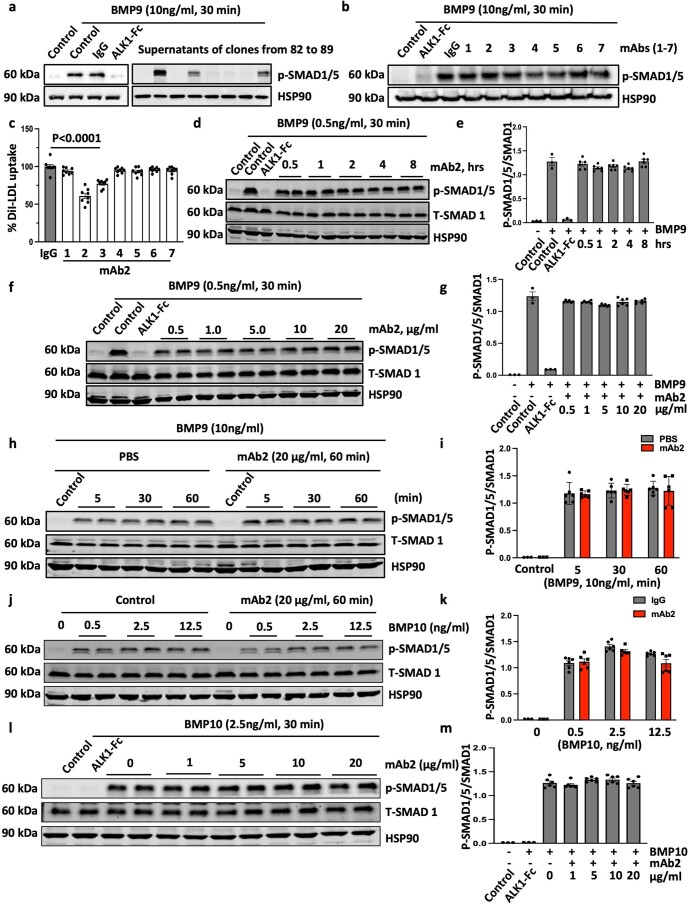
Selective, anti-ALK1 mAb screening and evaluation. **a**, A representative experiment showing how mAbs were screened. HUVEC were stimulated with BMP-9 (30 min) and the levels of p-SMAD1/5 were used as a readout for BMP-9 signaling. BMP9: 10 ng/ml, IgG: 20 μg/ml, ALK1-Fc: 1 μg/ml, supernatant concentration: 20 μg/ml. **b**, Western blot analysis showing that seven purified mAbs did not block BMP-9 stimulated p-SMAD1/5 signaling in HUVEC. BMP9: 10 ng/ml, IgG: 20 μg/ml, ALK1-Fc: 1 μg/ml, antibodies: 20 μg/ml. **c**, mAbs 2 and 3 reduced DiI-LDL uptake in HUVEC. IgG or mAb (20 μg/ml of each) was pre-incubated. Three independent experiments were performed. DiI-LDL fluorescence was quantified by Image J software. P < 0.0001 and Values show mean ± S.E.M. *P* values were calculated by two-way ANOVA with Tukey’s multiple comparisons test. **d,e**, Western blot (**d**) and quantification (**e**) showing that incubation with mAb for different time points (0.5-8hrs) did not impact BMP-9 / p-SMAD1/5 signaling. BMP9: 10 ng/ml, ALK1-Fc: 1 μg/ml. n = 3 for two controls and ALK1-Fc, and n = 6 for BMP9 treated samples. **f,g**, Western blot analysis (**f**) and quantification (**g**) showing that increasing doses of mAb2 (0.5-20μg/ml) does not impact BMP-9 signaling. BMP9: 10 ng/ml, ALK1-Fc: 1 μg/ml. n = 3 for two controls and ALK1-Fc, and n = 6 for BMP9 treated samples. **h,i**, Western blot (**h**) and quantification (**i**) showing that mAb2 does not block short term BMP-9 signaling. BMP9: 10 ng/ml, mAb2: 20 μg/ml. n = 3 for 0.5 min. and n = 6 for 5, 30, 60 min. BMP9 treatment. **j,k**, Western blot analysis (**j**) and quantification (**k**) showing that increasing doses of BMP10 (0.5-12.5 ng/ml) does not impact p-SMAD1/5 signaling in HUVEC pre-incubated with mAb2 (20 μg/ml for 60 min.). n = 3 for 0 and n = 6 for 0.5, 2.5, and 12.5 ng/ml of BMP10 treatment. **l,m**, Western blot analysis (**l**) and quantification (**m**) showing that increasing doses of mAb2 (1-20 μg/ml) does not impact BMP-10 signaling. BMP10: 2.5 ng/ml for 30 min. n = 3 for control and ALK1-Fc treatment and n = 6 for 0.5, 1, 5, 10, and 20 μg/ml mAb2 treatment. All experiments were repeated at least three times. Values show mean ± S.E.M. *P* values were calculated by two-way ANOVA with Tukey’s multiple comparisons test. Source data

**Extended Data Fig. 6:**
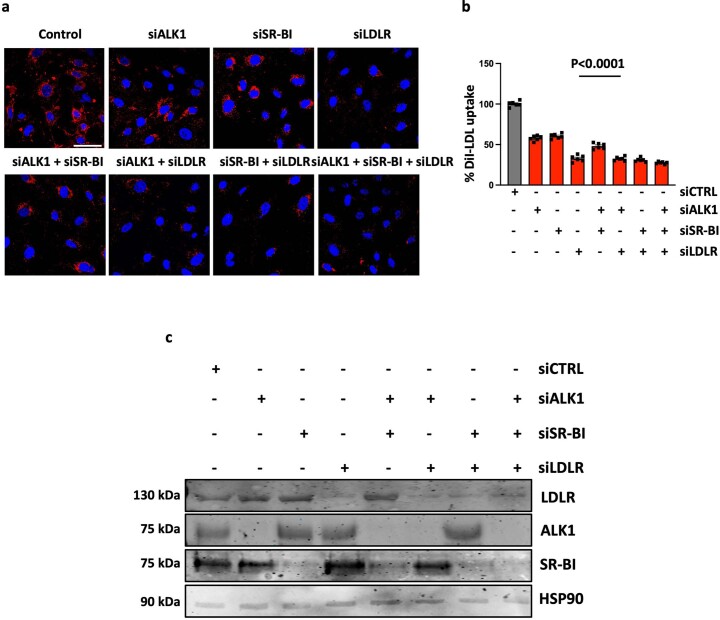
LDL uptake is regulated by ALK1, SR-BI, or LDLR. **a,b**, Representative confocal images (**a**), and quantification (**b**) of DiI-LDL uptake upon individual or pairwise silencing of ALK1, SR-BI, or LDLR in HUVEC. Two images were counted per sample in three separate experiments for a total of n = 6. Scale bar: 20μm. P < 0.0001 for b. Values show mean ± S.E.M. *P* values were calculated by one-way ANOVA with Sidak’s multiple comparisons test. **c**, Western blot showing knockdown efficiency of each ALK1, SR-BI, or LDLR. Three separate experiments were performed to confirm the knockdown. Source data

**Extended Data Fig. 7:**
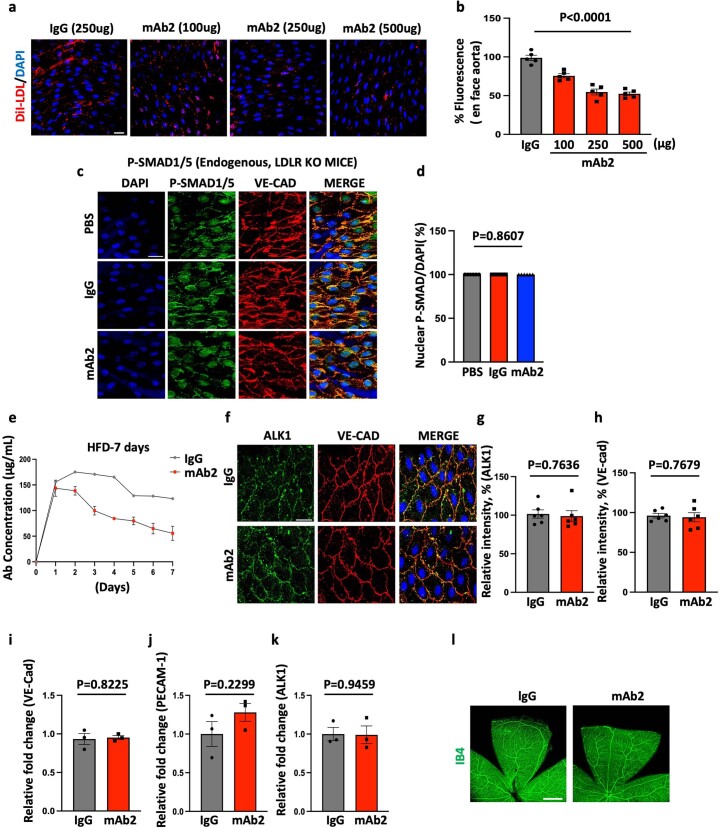
mAb2 concentration is reduced to half in 4 days and does not inhibit BMP-9 signaling or vascular tone in vivo. **a,b**, Representative en face images (**a**) and quantification (**b**) of DiI-LDL in *Ldlr*^−*/*−^ mouse aorta injected with either IgG or increasing doses of mAb2 (100-500 μg/mouse). One image was counted per mouse in five mice per group for a total of n = 5. Scale bar: 20μm. Image J software was used to quantify the samples. P < 0.0001 for b. Values show mean ± S.E.M. *P* values were calculated by one-way ANOVA with Sidak’s multiple comparisons test. **c,d**, Representative en face immunofluorescence images (**c**) and quantification (**d**) of endogenous p-SMAD1/5 localization in aortic arch of *Ldlr*^−*/*−^ mice injected with PBS, IgG, or mAb2. P = 0.8607 for d. Values show mean ± S.E.M. *P* values were calculated by one-way ANOVA with Sidak’s multiple comparisons test. Scale bar: 20μm. **e**, Serum antibody concentrations were measured using an ELISA assay. Anti-ALK mAb concentration change over seven days after single injection (250 μg) to *Ldlr*^−*/*−^ mice fed a Western diet for 7 days. Values show mean ± S.E.M. **f-h**, Representative confocal images (**f**), and analysis of ALK1 (**g**) and VE-cad intensity (**h**). Two images were counted per mouse in three mice per group for a total of n = 6. Scale bar: 20μm. P = 0.7636 for g and P = 0.7679 for h. Values show mean ± S.E.M. *P* values were calculated by two-tailed unpaired t-test. Image J software was used to quantify the ALK1 and VE-cad intensity. **i-k**, Relative VE-cad (**i**), PECAM-1 (**j**), and ALK1 (**k**) expression from isolated aortic ECs of *Ldlr*^−*/*−^ mice fed a Western diet for 6 weeks with IgG or mAb2 treatment. n = 3. P = 0.8225 for i, P = 0.2299 for j, and P = 0.9459 for k. Values show mean ± S.E.M. *P* values were calculated by two-tailed unpaired t-test. **l**, Isolectin B4 staining of retinal vessels of *Ldlr*^−*/*−^ mice fed a Western diet for 12 weeks with either IgG or mAb2 treatment. n = 6. Source data

BMP9 or BMP10 is a the cognate ligand that binds to ALK1 and this pathway is important for a wide range of diseases from pulmonary hypertension to cancer^19^. Previous work indicated that LDL can directly bind to ALK1 independent of its intrinsic kinase activity or coreceptors required for signaling^2^. Thus, we sought to develop a selective monoclonal antibody that blocks LDL binding to ALK1 but does not influence BMP signaling. Rodents^20^ carrying humanized immunoglobulin (Ig) loci were genetically immunized with cells expressing human ALK1 and monoclonal antibody isolated and screened against human, murine and monkey ALK1 and counter screened against human ALK5, using flow cytometry of cells expressing ALK1 or ALK5 for antibody selection. After initial screening, hybridoma supernatants of 89 candidate antibodies were screened in vitro using BMP9 stimulation of phospho-SMAD 1/5 (p-SMAD 1/5) signaling in human umbilical vein endothelial cells (HUVECs) as an assay (Extended Data Fig. 5a) and seven antibodies were confirmed to bind ALK1 but not block BMP9 signaling (Extended Data Fig. 5b). It is interesting that two monoclonal antibodies that bind ALK1 reduced LDL uptake into HUVECs (Extended Data Fig. 5c). Further testing showed that the monoclonal antibody mAb2 did not block BMP9 or BMP10 signaling regardless of concentration and incubation time of either BMP9/10 or mAb2 (Fig. 2a and Extended Data Fig. 5d–m). To examine direct interaction between mAb2 antibody and ALK1-Fc, surface plasmon resonance (SPR) was used with ALK-Fc immobilized on the chip and purified monoclonal anti-ALK1 antibody as the analyte. The SPR result showed high affinity of mAb2 to immobilized human ALK1 ectodomain, with an apparent dissociation constant, *K*_d_, of 5 ± 2 nM (Fig. 2b). Although mAb2 did not block BMP signaling (see above data) we assessed the direct protein–protein interactions via SPR. To test whether BMP9 or BMP10 and mAb2 compete for binding to ALK1-Fc, immobilized ALK1-Fc was saturated with BMP9 or BMP10 followed by injection of a saturating amount of mAb2. The mAb2 was still able to bind to ALK1-Fc after pretreatment with saturating amounts of BMP9 or BMP10 (Fig. 2c,d). Importantly, mAb2 dose-dependently blocked DiI-LDL uptake into HUVECs (Fig. 2e) and the expression of ALK1 was required because silencing of ALK1 abrogated the effect of the monoclonal antibody to block LDL uptake (Fig. 2f) to levels seen with silencing of ALK1 alone. The uptake of LDL in the absence of ALK1 in EC cultures is probably mediated by other LDL-binding proteins such as LDLR, SR-B1 or other proteoglycans that can mediate LDL uptake in HUVECs^21^. DiI-LDL uptake was reduced to 60% when ALK1, SR-BI or LDLR was individually silenced and pairwise or triple silencing of the genes further synergistically reduced DiI-LDL uptake (Extended Data Fig. 6a,b). Silencing of each gene was confirmed by western blotting (Extended Data Fig. 6c). To separate LDL uptake from LDL transcytosis, we quantified LDL transcytosis using total internal reflection fluorescence (TIRF) microscopy to selectively visualize the basolateral delivery of DiI-LDL^22^. Treatment of human coronary artery endothelial cells (HCAECs) with mAb2 markedly reduced LDL transcytosis (Fig. 2g). As initial candidates were screened against several species, mAb2 successfully reduced DiI-LDL uptake into mouse lung endothelial cells (MLECs; Fig. 2h). In addition, increasing single doses of mAb2 blocked DiI-LDL uptake into the endothelium of mouse aortas from *Ldlr*^−*/*−^ mice in vivo, showing 250 μg per mouse to be the maximal efficacious dose at this time point (Extended Data Fig. 7a,b); 250 μg per mouse of mAb2 did not block BMP9 signaling in the aortic arch of *Ldlr*^−*/*−^ mice (Extended Data Fig. 7c,d), as quantified by p-SMAD 1/5 in the ECs. Collectively, we successfully developed a selective monoclonal antibody against endogenous ALK1 that specifically blocks LDL uptake and transcytosis but not BMP9 signaling.

### Anti-ALK1 antibody inhibits atherosclerosis initiation and progression

**Fig. 3:**
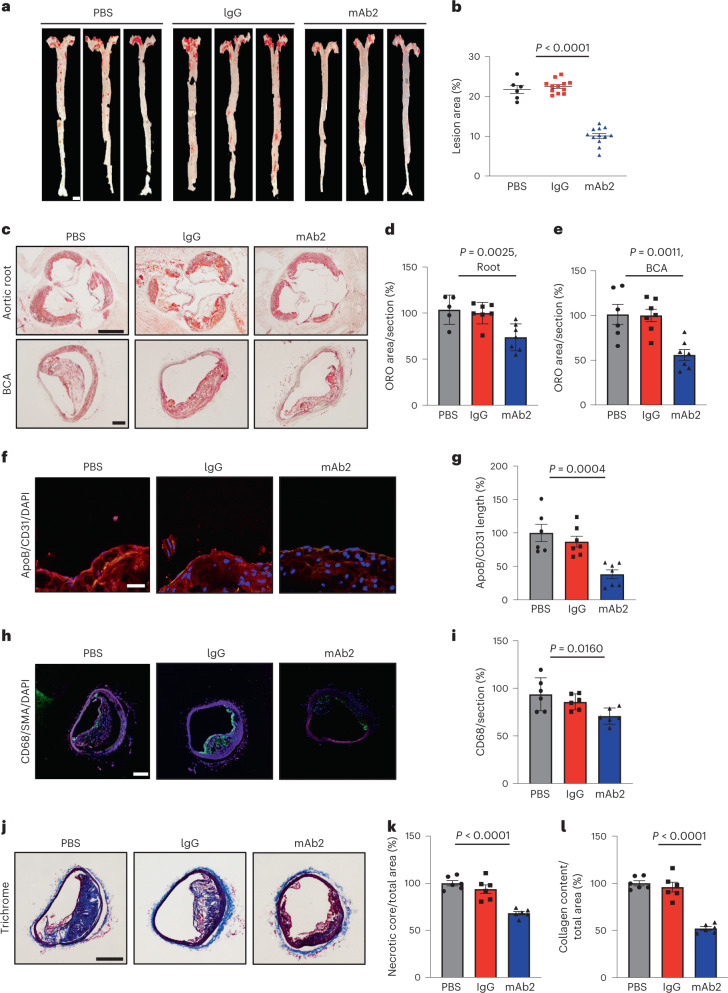
Efficient inhibition of apoB accumulation and atherosclerosis progression by mAb2. **a**,**b**, Representative images (**a**) and analysis of neutral lipids (**b**) by ORO staining in whole aorta. Scale bar, 2 mm. *P* < 0.0001. Values show mean ± s.e.m. **c**–**e**, Representative histological images (**c**) and analysis of neutral lipids by ORO in aortic roots (**d**) and BCAs (**e**). Scale bars, 500 μm for aortic root and 100 μm for BCA. *P* = 0.0025 for **d** and *P* = 0.0011 for **e**. Values show mean ± s.e.m. **f**,**g**, Confocal images (**f**) and analysis of apolipoprotein B (apoB) content per CD31 length per section (**g**) in the lesser curvature region of the aortic arch: red: apoB; green: CD31; blue: nuclei. Scale bar, 20 μm. *P* = 0.0004 for **g**. Values show mean ± s.e.m. **h**,**i**, Confocal images (**h**) and analysis of macrophage infiltration (**i**) in BCA sections: green: CD68; blue: nuclei. Scale bar, 100 μm. *P* = 0.0160 for **i**. Values show mean ± s.e.m. **j**–**l**, Representative histological images (**j**) and analysis for necrotic core (**k**) and collagen content (**l**) by trichrome staining in BCAs. Scale bar, 100 μm. *P* < 0.0001 for both **k** and **l**. All samples are from *Ldlr*^−*/*−^ mice treated with PBS, IgG or mAb2 fed a WD for 12 weeks. Mice were injected with 250 μg of IgG or mAb2 twice weekly during the 12-week feeding period. Aortic root and BCAs were sectioned at 6 μm (*n* = 12 mice). Values show mean ± s.e.m. *P* values were calculated by two-way ANOVA with Tukey’s multiple comparisons test. Source data

**Extended Data Fig. 8:**
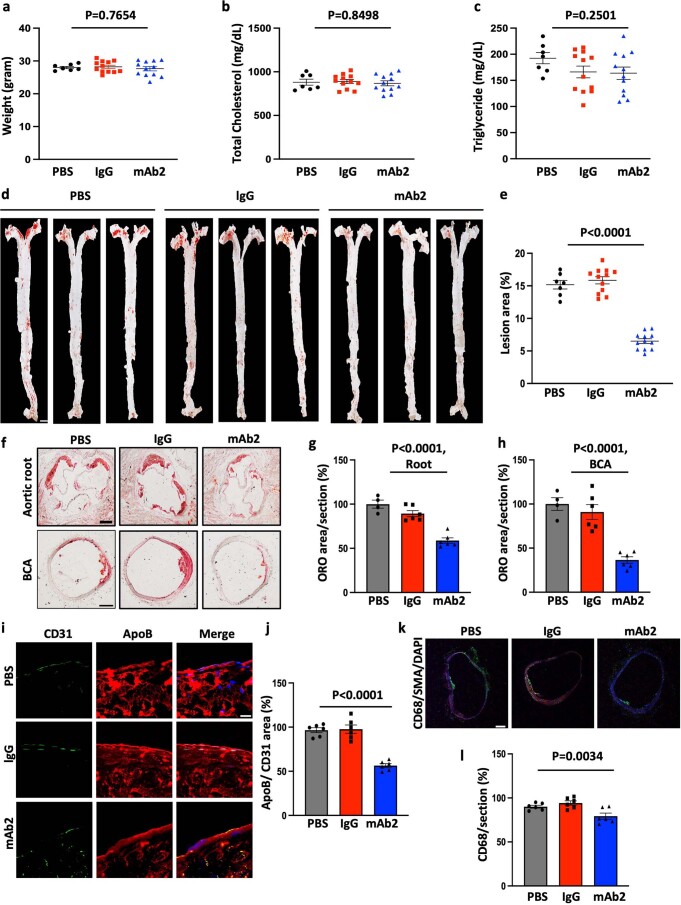
Selective anti-ALK mAb2 attenuates the initiation of atherosclerosis in mice. **a-c**, mAb2 did not impact the weight (**a**) or plasma lipids (total cholesterol (**b**) and triglycerides (**c**)) of *Ldlr*^−*/*−^ mice fed a Western diet for 6 weeks. n = 7 for PBS treatment and n = 12 for IgG and mAb2 treated mice. P = 0.7654 for a, P = 0.8498 for b, and P = 0.2501 for c. Values show mean ± S.E.M. **d,e**, Individual aorta images (**d**) and quantification (**e**) of neutral lipids from *Ldlr*^−*/*−^ mice fed a Western diet for 6 weeks. Total 250 μg of IgG or mAb2 was injected twice weekly for 6 weeks duration. n = 7 for PBS treatment and n = 12 for IgG and mAb2 treated mice. Scale bar: 2 mm. Image J software was used to quantify the ORO area. P < 0.0001 for e. Values show mean ± S.E.M. *P* values were calculated by one-way ANOVA with Sidak’s multiple comparisons test. **f-h**, Representative images (**f**), and quantification showing ORO staining of aortic root (**g**) and brachiocephalic arterial sections (**h**). Scale bars: 500μm and 100μm, for aortic root and brachiocephalic artery, respectively. P < 0.0001 for both g and h. Values show mean ± S.E.M. **i,j**, Representative confocal images (**i**) and analysis (**j**) showing apoB (red) content per CD31 length/section of brachiocephalic artery. Scale bar: 20μm. P < 0.0001 for j. Values show mean ± S.E.M. **k,l**, Confocal images (**k**) and analysis (**l**) showing macrophage infiltration, green:CD68. Scale bar: 100μm. All samples are from *Ldlr*^−*/*−^ mice treated with PBS, IgG, or mAb2 and western diet for 6 weeks. Total 250 μg of IgG or mAb2 was injected twice per week for 6 weeks. Aortic root and brachiocephalic arteries were sectioned at 6μm. P = 0.0034 for l. Values show mean ± S.E.M. *P* values were calculated by one-way ANOVA with Sidak’s multiple comparisons test. Source data

To determine whether mAb2 could be exploited as a translational tool impacting atherosclerosis, three cohorts of *Ldlr*^−*/*−^ mice were fed a WD for either 6 weeks (initiation study) or 12 weeks (progression study) and administered phosphate-buffered saline (PBS), rat IgG1 or anti-ALK1 mAb2. For in vivo studies, 250 μg per mouse of IgG1 or mAb2 was injected twice weekly because half the amount of mAb2 concentration remained in the serum of *Ldlr*^−*/*−^ mice after 4 d (Extended Data Fig. 7e). Bi-weekly injection of mAb2 for 6 weeks did not change aortic ALK1, VE-cad or PECAM-1 levels (Extended Data Fig. 7f–k) or induce vascular malformation, as examined in retinal vessels (Extended Data Fig. 7l). In the initiation study, no changes in weight and lipid parameters were observed between PBS- and IgG- and mAb2-injected *Ldlr*^−*/*−^ mice (Extended Data Fig. 8a–c), but mAb2 treatment was associated with a marked reduction in plaque formation in aorta (Extended Data Fig. 8d,e), aortic roots and the BCA compared with PBS or IgG treatment (Extended Data Fig. 8f–h). The reduction in lesion size was accompanied by a decrease in apoB content and CD68^+^ macrophages in the lesions (Extended Data Fig. 8i–l). Similarly, in the progression study, mAb2 did not alter body weight, plasma lipids or blood glucose (Extend Data Fig. 9a–d), but markedly reduced the extent of atherosclerosis in the aorta (Fig. 3a,b), aortic roots and the BCA (Fig. 3c–e) and reduced the accumulation of apoB-containing lipoproteins (Fig. 3f,g), CD68^+^ macrophages (Fig. 3h,i), plaque necrosis (Fig. 3j,k) and collagen content (Fig. 3l). Treatment with mAb2 for 12 weeks did not alter white blood cell, red blood cell or platelet counts (Supplementary Table 2). Thus, our data demonstrate that a selective anti-ALK1 monoclonal antibody reduces atherogenesis by inhibiting the accumulation of apoB-containing lipoproteins, thereby reducing plaque burden, inflammation and necrosis.

**Extended Data Fig. 9:**
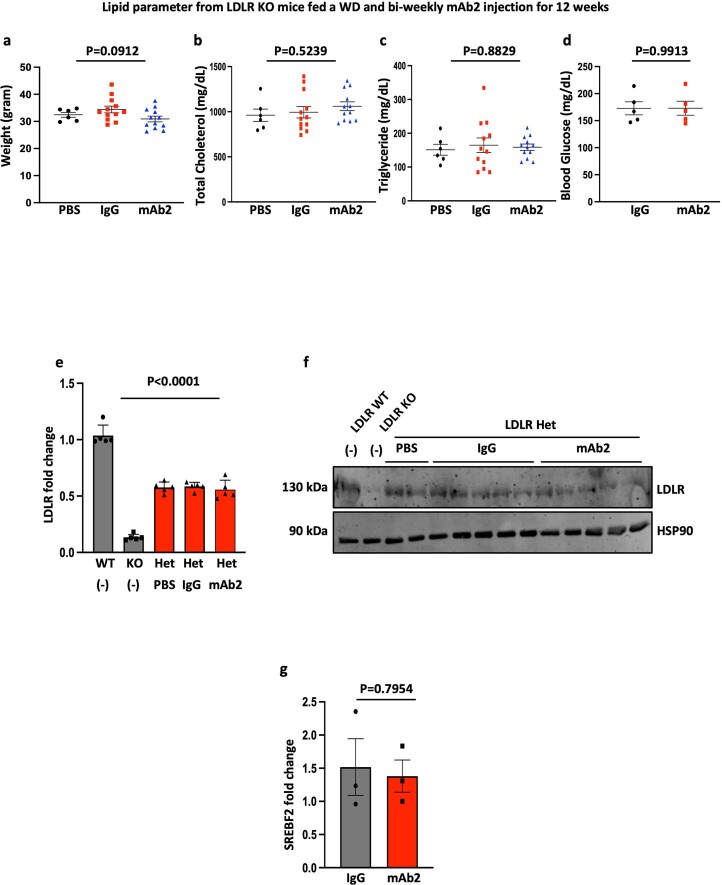
Selective anti-ALK mAb2 attenuates the progression of atherosclerosis in mice and mAb2 operates independently of LDL receptor in vivo. **a-d**, Analysis of weight (**a**), plasma total cholesterol (**b**), triglyceride (**c**), and blood glucose level (**d**) from *Ldlr*^−*/*−^ mice fed a western diet for 12 weeks with IgG or mAb2 treatment. Bi-weekly injections of 250 μg per injection. n = 12. P = 0.0912 for a, P = 0.5239 for b, P = 0.8829 for c, and P = 0.9913 for d. Values show mean ± S.E.M. *P* values were calculated by one-way ANOVA with Sidak’s multiple comparisons test. **e,f**, Relative mRNA (**e**) and protein LDLR (**f**) expression level from the livers of LDLR WT, KO, and Het mice fed a Western diet for 6 weeks with antibody treatment. n = 5. P < 0.0001 for e. Values show mean ± S.E.M. *P* values were calculated by one-way ANOVA with Sidak’s multiple comparisons test. **g**, Relative sterol-sensing gene, SREBF2, expression from the livers of LDLR WT, KO, and Het mice fed a Western diet for 6 weeks with antibody treatment. n = 3. P = 0.7954. Values show mean ± S.E.M. *P* values were calculated by two-tailed unpaired t-test. Source Data.

It is well appreciated that LDLR is suppressed during hyperlipidemia due to a well-described negative feedback loop and de-repression of hepatic LDLR levels results in enhanced cholesterol clearance, accounting for most of the benefit of statins and PCSK9 inhibitors. Although ALK1 was discovered as an LDLR-independent alternative pathway for LDL entry into ECs and characterized as being nondegradative for apoB100 and sterol insensitive, we wanted to glean a deeper mechanistic understanding of the relationship between ALK1 and sterol responsiveness of LDLR in vivo. To address this, *Ldlr*^−*/*−^ knockout (KO) or *Ldlr*^*+/*−^ (heterozygous) mice were given IgG or mAb2 with a WD for 6 weeks. Both LDLR mRNA and protein levels were reduced to half compared with wild-type whereas the levels were twice that of KO mice (Extended Data Fig. 9e,f). The mRNA levels of the major transcriptional regulator of sterol responsiveness, sterol responsive-binding protein 2 (*SREBF*), was the same between IgG- and mAb2-treated groups (Extended Data Fig. 9g), indicating that neutralization of ALK1 in the vessel does not promote compensation of the SREBPF2–LDLR axis in the liver. However, future experiments testing mAb2 in a model of hyperlipidemia with intact LDLR will be informative.

### Anti-ALK1 antibody accelerates atherosclerosis regression

**Fig. 4:**
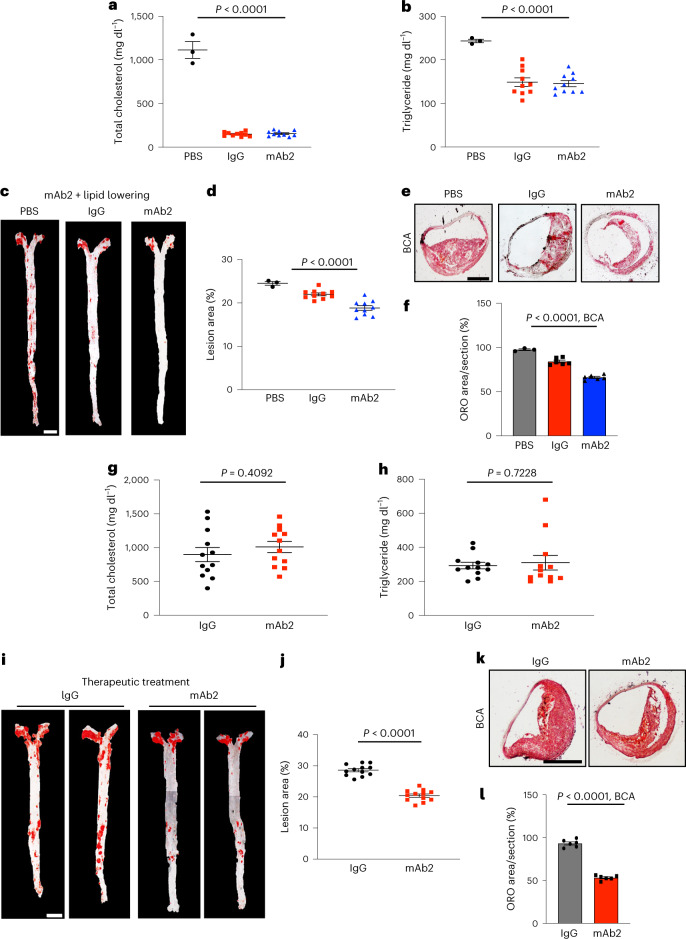
A combination of mAb2 with dietary lipid lowering synergistically reduces plaque formation and mAb2 treatment alone inhibits atherosclerosis progression during sustained hyperlipidemia. **a**,**b**, Plasma total cholesterol (**a**) and triglyceride (**b**) levels measured at endpoint of 16 weeks: 12 weeks of WD followed by 4 weeks of normal chow diet (*n* = 10 mice per group for IgG or mAb2 injected and *n* = 3 for diet alone). *P* < 0.0001 for both **a** and **b**. Values show mean ± s.e.m. **c**,**d**, Representative images (**c**) and analysis of whole aorta (**d**) showing accumulation of neutral lipids by ORO staining of *Ldlr*^−*/*−^ mice injected with IgG or mAb2 and fed a WD for 12 weeks followed by normal diet for 4 weeks (*n* = 10 mice per group). Scale bar, 2 mm. *P* < 0.0001 for **d**. Values show mean ± s.e.m. **e**,**f**, Representative ORO-stained images (**e**) and quantification of neutral lipid content (**f**) of BCAs (*n* = 3 for PBS- and *n* = 6 for IgG- and mAb2-treated BCA). Scale bar, 100 μm. *P* < 0.0001 for **f**. Values show mean ± s.e.m. **g**,**h**, Plasma total cholesterol (**g**) and triglyceride (**h**) levels after 16 weeks of WD and, IgG or mAb2 was injected during the last 4 weeks (*n* = 12 mice per group). *P* = 0.4902 for **g** and *P* = 0.7228 for **h**. Values show mean ± s.e.m. **i**,**j**, Representative images (**i**) and analysis of whole aorta (**j**) showing accumulation of neutral lipids by ORO staining of *Ldlr*^−*/*−^ mice fed a WD for 16 weeks and IgG or mAb2 injected during the last 4 weeks (*n* = 12 mice per group). Scale bar, 2 mm. *P* < 0.0001 for **j**. Values show mean ± s.e.m. **k**,**l**, Representative ORO-stained images (**k**) and quantification of neutral lipid content (**l**) of BCAs (*n* = 6). Scale bar, 100 μm. *P* < 0.0001 for **j**. Value shows mean ± s.e.m. All BCAs were sectioned at 6 μm. *P* values were calculated by two-way ANOVA with Tukey’s multiple comparisons test for **a**–**f** and by two-tailed, unpaired Student’s *t*-test for **g**–**l**. Source data

**Extended Data Fig. 10:**
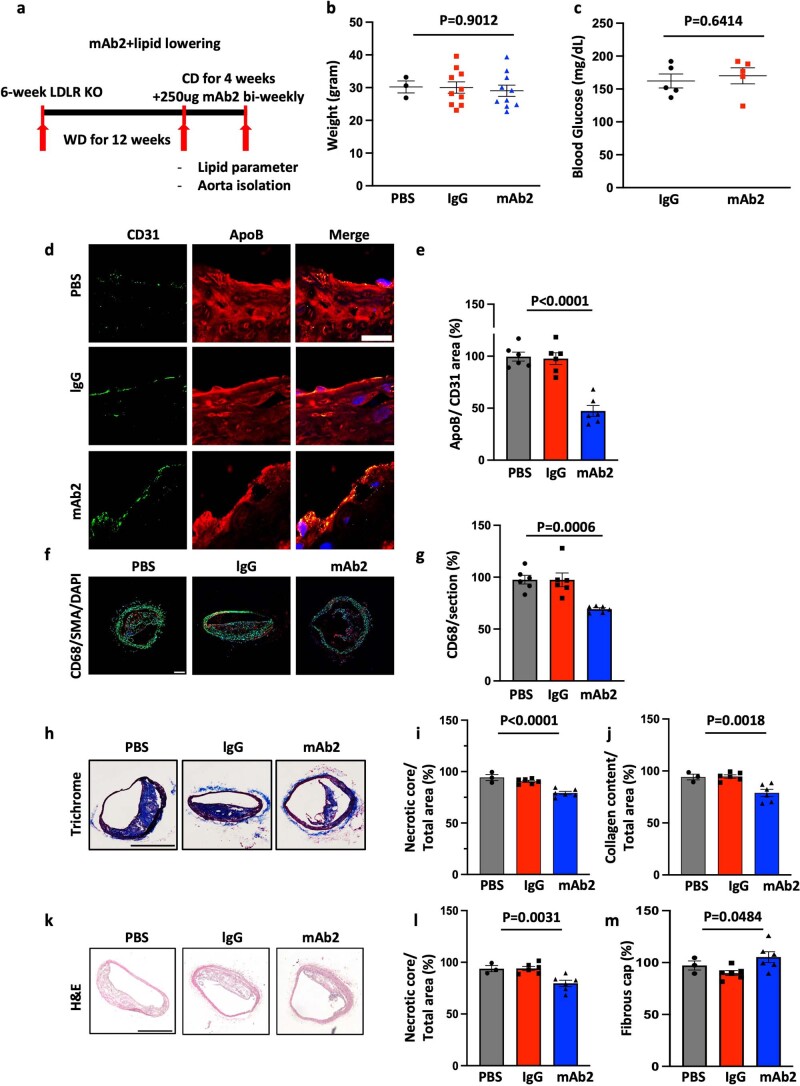
mAb2 treatment in lipid lowering condition further reduces the progression of atherosclerosis. **a**, Schematic diagram showing experimental procedure. Samples are prepared from *Ldlr*^−*/*−^ mice fed a Western diet for 12 weeks with another 4 weeks of normal diet. The mice received IgG or mAb2 during the last 4 weeks, twice weekly with 250μg per injection. Control mice were sacrificed after 12 weeks of Western diet while IgG or mAb2 treated mice were sacrificed after 16 weeks. **b,c**, Analysis of weight (**b**)and blood glucose (**c**) level from *Ldlr*^−*/*−^ mice fed a Western diet for 12 weeks with another 4 weeks of normal diet to lower lipid. The mice received twice weekly injections of 250 μg per injection during the last 4 weeks. n = 10. P = 0.9012 for b and P = 0.6414 for c. Values show mean ± S.E.M. **d,e**, Representative confocal images (**d**), and analysis (**e**) of brachiocephalic artery sections for apoB content, red: apoB, green: CD31, blue: nuclei. Scale bar: 20μm. P < 0.0001 for e. Values show mean ± S.E.M. **f,g**, Confocal images (**f**) and analysis (**e**) of brachiocephalic arterial sections for macrophage infiltration, green: CD68, blue: nuclei. Scale bar: 100μm. P = 0.0006 for g and Values show mean ± S.E.M. **h-j**, Trichrome staining (**h**) of brachiocephalic artery sections for plaque necrosis (**i**) and collagen content (**j**). Scale bar: 100μm. P < 0.0001 for i and P = 0.0018 for j. Values show mean ± S.E.M. **k-m**, H&E staining (**k**) of brachiocephalic artery sections for plaque necrosis (**l**) and fibrous cap thickness (**m**). Scale bar: 100μm. All aortic root and brachiocephalic arteries were sectioned at 6μm. n = 6 mice per group. Image J software was used to quantify the samples. P = 0.0031 for l and P = 0.0484 for m. Values show mean ± S.E.M. *P* values were calculated by one-way ANOVA with Sidak’s multiple comparisons test. Source data

**Extended Data Fig. 11:**
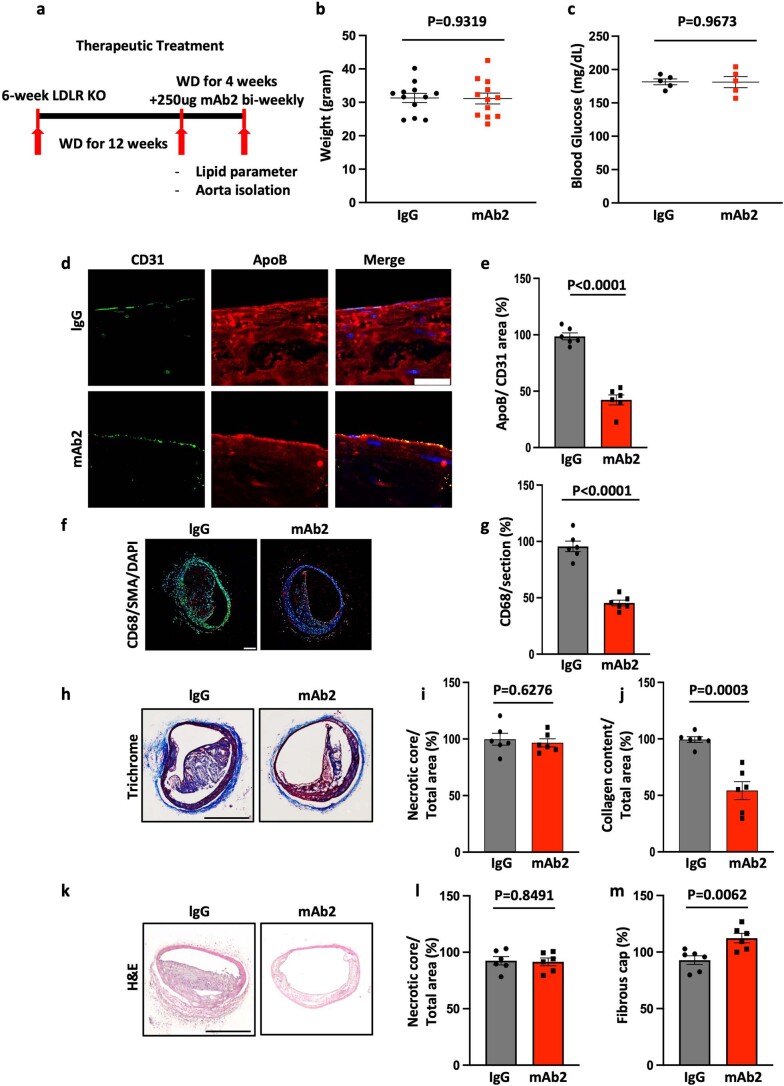
Therapeutic treatment of mAb2 reduces atherosclerosis progression. **a**, Schematic diagram showing experimental procedure. Samples are prepared from *Ldlr*^−*/*−^ mice fed a Western diet for 16 weeks. The mice received IgG or mAb2 during the last 4 weeks, twice weekly with 250μg per injection. **b,c**, Analysis of weight (**b**) and blood glucose (**c**) level from *Ldlr*^−*/*−^ mice fed a Western diet for 16 weeks. The mice received twice weekly injections of 250 μg per injection during the last 4 weeks. n = 12. P = 0.9319 for b and P = 0.9673 for c. Values show mean ± S.E.M. *P* values were calculated by one-way ANOVA with Sidak’s multiple comparisons test. **d,e**, Representative confocal images (**d**), and analysis (**e**) of brachiocephalic artery sections for apoB content, red: apoB, green: CD31, blue: nuclei. Scale bar: 20μm. P < 0.0001 for e. Values show mean ± S.E.M. **f,g**, Confocal images (**f**) and analysis (**g**) of brachiocephalic arterial sections for macrophage infiltration, green: CD68, blue: nuclei. Scale bar: 100μm. P < 0.0001 for g. Values show mean ± S.E.M. **h-j**, Trichrome staining (**h**) of brachiocephalic artery sections for plaque necrosis (**i**) and collagen content (**j**). Scale bar: 100μm. P = 0.6276 for i and P = 0.0003 for j. Values show mean ± S.E.M. **k-m**, H&E staining (**k**) of brachiocephalic artery sections for plaque necrosis (**l**) and fibrous cap thickness (**m**). Scale bar: 100μm. All aortic root and brachiocephalic arteries were sectioned at 6μm. n = 6 mice per group. Image J software was used to quantify the samples. P = 0.8491 for l and P = 0.0062 for m. Values show mean ± S.E.M. All *P* values were calculated by two-tailed unpaired t-test. Source data

Next, we assessed whether neutralization of ALK1 would be additive to lipid-lowering approaches and whether this mechanism would be beneficial therapeutically in a sustained model of familial hypercholesterolemia. As most mouse models of atherosclerosis are nonresponsive to statins and PCSK9 inhibitors due to the critical role of the LDLR in their therapeutic mechanisms^23–25^, we performed lesion regression studies in LDLR KO mice. *Ldlr*^−*/*−^ mice aged 6 weeks were fed a WD for 12 weeks to establish lesions, followed by switching the diet to normal chow for an additional 4 weeks (Extended Data Fig. 10a). In this model, the reduction in plasma cholesterol permits detection of lesion regression or lack of progression after 4 weeks of lipid lowering. Thus, three groups of *Ldlr*^−*/*−^ mice were fed a WD for 12 weeks, a small cohort sacrificed at 12 weeks and the remaining mice randomized into IgG versus mAb2 treatment groups (250 μg per mouse bi-weekly) for an additional 4 weeks while on normal chow. Switching the diet for 4 weeks markedly lowers plasma cholesterol and triglyceride levels, resulting in lesion regression/lack of progression (Fig. 4a,b). Four weeks of mAb2 treatment under lipid-lowering conditions reduced atherosclerosis in en face aorta (Fig. 4c,d) and the BCA (Fig. 4e,f), and apoB content, macrophage infiltration, plaque necrosis, collagen content and fibrous cap thickness in the BCA (Extended Data Fig. 10d–m). Finally, we assessed whether mAb2 would impact established lesion progression. *Ldlr*^−*/*−^ mice were fed a WD for 12 weeks to establish lesions followed by mAb2 treatment for an additional 4 weeks (Extended Data Fig. 11a). Antibody treatments did not change weight, blood glucose (Extended Data Fig. 11b,c), total cholesterol or triglyceride levels (Fig. 4g,h), but mAb2 substantially reduced plaque formation in the aorta (Fig. 4i,j). In BCA sections, mAb2 treatment demonstrated a dramatic decrease in lipid accumulation (Fig. 4k,l), apoB content, macrophage infiltration, plaque necrosis, collagen content and fibrous cap thickness in the BCA (Extended Data Fig. 11d–m).

In summary the uptake and retention of apoB-containing lipoproteins into the vessel wall is well appreciated as a key event in the genesis of atherosclerosis, as well as a surrogate of plague regression after lipid lowering^1,7,26^. For decades, LDL movement into the vessel wall was thought to be passive, occurring at atheroprone regions of the vessel wall that exhibit enhanced permeability for the movement of plasma proteins into blood vessels. Our study shows that ALK1-dependent LDL entry into the endothelium is a critical step for apoB entrapment in the vessel wall and provides a therapeutic rationale to block LDL entry into the endothelium as a mechanism to treat the vessel wall and alter the course of ASCVD. Theoretically this approach could be combined with traditional lipid-lowering therapies to improve vascular health.

## Methods

No statistical methods were used to predetermine sample size, but were based on historical numbers of mice used in the atherosclerosis field^27^. All the experiments were blinded for tissue analysis. In experiments examining the effects of mAb2 on atherosclerosis, the main author was aware of mice receiving injections of PBS, IgG and mAb2; however, specimens were analyzed in a blinded manner by an additional scientist.

### Gene expression profiling in human atherosclerotic versus normal arteries

To evaluate ALK1 expression in human atherosclerotic versus normal arteries, a publicly available human atherosclerosis cohort with gene expression data was downloaded from Gene Expression Omnibus^28,29^ (GEO, https://www.ncbi.nlm.nih.gov/geo), accession no. GSE43292. For the cohort, gene expression profiling was generated by Affymetrix APT package (http://media.affymetrix.com/support/developer/powertools/changelog/apt-probeset-summarize.html). RMA algorithm (default parameter setting) was applied to process CEL files and then APT package was applied to summarize the gene expression results. The atherosclerotic carotid artery and nonatherosclerotic area of the healthy artery were subject matched. Data are presented as scatter plots.

### ScRNA-seq analysis

The scRNA-seq datasets were obtained from a previously published paper and analyzed from three AC regions and three PA regions^30^. The unbiased clustering in the dataset identified three subpopulations of endothelial cells (clusters 2, 9 and 15). The datasets are available in the GEO (http://pubmed.ncbi.nlm.nih.gov/36224302) with accession no. GSE159677.

#### Animal models

Experiments were performed only with male *Alk1*^*fl/*fl^*BmxCre*^*ERT2*^*mTmG*^13^, *Alk1*^*fl/fl*^*BmxCre*^*ERT2*^*Ldlr*^−*/*−^, *Ldlr*^−*/*−^ mice and *Ldlr*^*+/*−^ mice, all congenic on a *Mus musculus* C57BL/6 genetic background. At 6 weeks of age (3–12 mice), littermate male mice were injected with single 1.5 mg of tamoxifen intraperitoneally to delete the *Alk1* allele. Atherosclerosis was induced by feeding the mice a WD (Research Diets, Inc., catalog no. D120108) for 4, 12 and 16 weeks, respectively. Mice used in all experiments were sex and age matched and kept in individually ventilated cages in pathogen-free rooms. Mice were housed in the Yale Animal Facility, fed normal chow (Harlan Teklad, catalog no. rodent diet 2018) and kept at 25 °C on a 12-h light:dark cycle. The Institutional Animal Care and Use Committee (IACUC) of Yale University approved all the experiments. All animals were handled according to the IACUC protocol (no. 07919-2020) of Yale University.

### Human specimen collection

Research protocols were approved by the Institutional Review Boards of Yale University (protocol no. 2023-07995). A waiver for consent was approved for surgical patients and written informed consent was obtained from a member of the family for a deceased organ donor. Investigators were on call with the surgical team and collected the heart at the time of explant. To minimize ex vivo artifacts, an approximately 5- to 20-mm segment of the left main coronary artery was removed within the operating room and immediately processed as frozen sections in optimal cutting temperature (OCT) medium (Sakura Finetek USA, Inc.) and, when of sufficient length, an additional segment was also fixed in formalin for later embedding, sectioning and staining^31^. Serial sectioning of representative samples of de-identified specimens of nonatherosclerotic, mildly, moderately or severely atherosclerotic vessels was used for immunofluorescent imaging in Extended Data Fig. 1a.

### Mammalian cell culture

HUVECs were obtained from the Yale School of Medicine, Vascular Biology and Therapeutics Core facility. HCAECs were obtained from Promocell (catalog no. C-12221). Cells were cultured in EGM-2 medium (Lonza) with 5% fetal bovine serum, penicillin–streptomycin and glutamine (2.8 mM) in a 37 °C incubator with 5% CO_2_ supply.

#### Atherosclerosis evaluation and lipid analyses

Atherosclerotic lesions were evaluated as previously described^32,33^. Mice were anaesthetized with isoflurane, blood was collected for lipid analyses and the vascular system was perfused with 4% paraformaldehyde (PFA), administered by left ventricular puncture. Adventitial fat was removed from the aortic arch before ORO imaging. The heart and whole aorta were isolated and placed in 4% PFA for fixation overnight; the heart was dehydrated in 30% sucrose at 4 °C overnight and embedded in OCT compound (Fisher Healthcare), and serial frozen sections (6 μm) of the aortic root and BCA were obtained. Then, 10–15 slides with 6 sequential sections each were prepared from each heart and BCA, and 1–2 slides per heart were processed for ORO/apoB/CD31/CD68/Trichrome staining of the aortic root and the BCA. For en face analysis, the entire aorta from the aorta root through the bifurcation of the iliac arteries was stained with ORO for 2 h at room temperature (RT), adjoining tissues were removed and the aorta was opened longitudinally and pinned on to a black silicon bed. Lesions were quantified by morphometry of obtained images using Photoshop software.

#### Lipids and lipoprotein profile

Lipid parameters were measured as previously described^34^. Briefly, mice were fasted for 12 h before blood samples were collected. Then, plasma from the samples was separated by centrifugation at 4 °C for 15 min and stored at −80 °C if necessary. Total plasma cholesterol and triglycerides were enzymatically measured according to the manufacturer’s instructions (Wako Pure Chemicals Tokyo, catalog nos. TC: 999-02601 and TG: 992-02892).

### Antibody generation

The human ALK1 complementary DNA was used for genetic immunization of rodent lines (Omnimouse, Omnirat lambda and Omnirat kappa) optimized to generate partially humanized monoclonal antibodies. Then, 300 hybridomas were generated and screened against human, mouse and monkey ALK1, and human ALK5 by flow cytometry in cells expressing the respective cDNAs. Some 89 candidates were determined to bind the 3 species but not human ALK5. Supernatant of each of the 89 candidates was tested for SMAD 1/5 phosphorylation by BMP9 treatment in HUVECs. Out of 89 candidates, 7 samples were determined not to block p-SMAD 1/5 signaling. The seven antibodies were purified and tested for further validation in p-SMAD 1/5 signaling and LDL transcytosis.

#### TIRF-based transcytosis of fluorescently labeled LDL

LDL transcytosis by confluent primary HCAECs was measured by TIRF microscopy^9,35^. Briefly, HCAECs were placed in a live cell chamber at 100% confluency and incubated with 20 μg ml^−1^ of either control IgG or mAb2 in 500 μl of serum-free medium at 37 °C for 2 h. Cells were washed 3× with cold PBS and treated with 20 μg ml^−1^ of DiI-LDL in cold HPMI (RPMI (Roswell Park Memorial Institute) growth medium with Hepes) at 4 °C for 10 min to allow apical membrane binding. Cells were washed 2× with cold PBS and at RT HPMI was added. The chamber was placed on a 37 °C live cell imaging stage for 2 min before imaging. Confluent regions of the monolayer were selected by viewing the number of nuclei in the DAPI field of view after staining with NucBlue Live ReadyProbes Reagent (Thermo Fisher Scientific) and TIRF images of the basal membrane were acquired on a Leica DMi8 microscope with: ×63/1.47 (O) objectives; 405-, 488-, 561- and 637-nm laser lines; 450/50, 525/50, 600/50, 610/75 and 700/75 emission filters; and run with Quorum acquisition software. Microscope settings were kept constant between conditions. A single-particle tracking algorithm developed in MATLAB (MathWorks, catalog no. R2014b) was used to quantify the number of transcytosis events by analyzing the videos.

### In vivo aorta LDL uptake

DiI-human nLDL was purchased from Kalen Biomedical, LLC (catalog no. 770230-9). Briefly, mice were injected with 100 μg of DiI-LDL via the tail vein^2,32,33^. After 30 min, mice were euthanized using ketamine (100 mg kg^−1^) and xylazine (10 mg kg^−1^). After anesthesia, the thoracic cavity was exposed and the aorta fixed with 4% PFA administered via the left cardiac ventricle. Aortas were isolated and subjected to Hoechst staining (5 μg ml^−1^) at 4 °C for 20 min to identify nuclei. Samples were washed 3× with 1× PBS and mounted on a glass slide. To test whether mAb2 could attenuate DiI-LDL uptake, mice were injected intraperitoneally with 300 μg of each control IgG or mAb2. After 1 h, DiI-LDL was injected via the tail vein. After 30 min, the aorta was perfused and isolated after Hoechst staining for 20 min. A confocal fluorescence microscope LEICA510 was used to obtain a minimum of three random images and quantified using an ImageJ program.

#### Blood pressure measurement

Arterial pressure in *Alk1*^*f/f*^*Ldlr*^−*/*−^ and *Alk1*^*iΔaEC*^*Ldlr*^−*/*−^ mice or *Alk1*^*f/f*^ and *Alk1*^*iΔaE*^ mice was measured by radiotelemetry. Briefly, the mice were anesthetized with a mixture of ketamine/xylazine (100 and 10 mg kg^−1^, respectively). A Data Sciences catheter was inserted into the carotid artery and a transmitter was placed in a subcutaneous pocket on the right flank^36^. Baseline values were continuously recorded every minute for 4 days beginning 1 week after surgery. Values were analyzed as 12-h means reflecting the day (6.30 a.m. to 6.30 p.m.) and night (6.30 p.m. to 6.30 a.m.) periods. RVSP was measured with a 1.4-F pressure transducer catheter (Millar Instruments) and Lab Chart software (ADInstruments). Briefly, mice were anesthetized with 2% isoflurane, then the catheter was inserted through the right jugular vein into the right ventricle.

### ORO staining

At the experimental endpoint, mice were perfused with PBS and then fixed with 4% PFA^2,32,33^. Using a dissecting microscope, whole aortas from the mice were isolated and the adventitial tissue was removed followed by fixation in 4% PFA for 1 h. Then, the samples were thoroughly rinsed in 78% methanol for 30 min and incubated in ORO solution (35 ml of 0.2% ORO in methanol with 10 ml of 1 M NaOH) for 1 h. The aorta was de-stained briefly with 78% methanol twice and washed with PBS. The aorta was cut longitudinally along the greater curvature and pinned open en face submerged in PBS. The sample images were captured and quantified using an ImageJ program.

#### DiI-LDL uptake

To evaluate DiI-LDL uptake, HUVECs were seeded on a round cover glass in 24-well plates. Next day, cells were incubated with 2.5 μg ml^−1^ of DiI-LDL for 30 min at 37 °C (ref. ^3^). Cells were washed 3× with 1× PBS and incubated with Hoechst for 15 min at RT. To test antibody efficacy, cells were incubated with 20 μg ml^−1^ for 30 min after DiI-LDL treatment (2.5 μg ml^−1^) at 37 °C for 30 min. Cells were then washed 3× with 1× PBS and stained by Hoechst at 37 °C for 10 min. The cover glass with cells was mounted on a glass slide and a confocal microscope was used to obtain randomly selected images and quantified using an ImageJ program.

#### Western blotting

Cells or tissues were lysed with ice-cold radioimmunoprecipitation assay buffer (RIPA) lysis buffer including phosphatase and protease inhibitor (Roche Diagnostics). A total of 20 μg of protein was loaded into NuPAGE 3–8% Tri Acetate Gel (Invitrogen): 10% sodium dodecylsulfate–polyacrylamide gel electrophoresis gel followed by transfer to nitrocellulose membranes. Western blotting was performed with the following primary antibodies: apoB (Meridian Life Science, Inc., catalog no. K23300R), HSP90 (BD Biosciences, catalog no. 610419), ALK1 (Fitzgerald, catalog no. 70R-49334) and p-SMAD 1/5 (Cell Signaling, catalog no. 9516).

#### Histology, immunohistochemistry and morphometric analyses

Mice were euthanized as above, the thoracic cavity was exposed and in situ perfusion was fixed with 4% PFA through the left ventricle. Mouse hearts, aortas and aortic arches were isolated, fixed overnight (O/N) in 4% PFA, then dehydrated with 30% sucrose O/N, embedded in OCT and frozen at −80 °C. For morphometric analysis, serial sections were cut at 6-μm thickness using a cryostat. Every third slide from the serial sections was stained with hematoxylin and eosin and each consecutive slide was stained with ORO for the quantification of the lesion area and lipid accumulation, respectively. Aortic lesion size was obtained by averaging the lesion areas in 3 slides per mouse, a total of 12 images. Snap-frozen 6-μm BCA sections from the aorta were used for immunofluorescence. Briefly, frozen aortic sections were fixed in 4% PFA for 10 min and then incubated at 4 °C O/N with primary antibodies for apoB-48/100 (Meridian Life Science, Inc., catalog no. K23300R), CD68 (AbD Serotec, catalog no. MCA1957) and CD31 (Abcam, catalog no. ab28364) after blocking with blocker buffer (5% donkey serum, 0.5% bovine serum albumin and 0.3% Triton X-100 in PBS) for 1 h at RT, followed by incubation with Alexa Fluor secondary antibody (Invitrogen) for 1 h at RT. The stained sections were captured using a LEICA510 confocal microscope and images were digitized under constant exposure time, gain and offset. Images were quantified as positive staining area (µm^2^) per length of the endothelial cell layer (µm) per section measured using the ImageJ (National Institutes of Health (NIH)) software.

### Antibodies

Primary antibodies were: anti-ALKl blocking (Genovac, not commercially available), ApoB (1:100, Meridian Life Science, Inc., catalog no. K23300R), HSP90 (1:500, BO Biosciences, catalog no. 610419), human ALKl (1:500, Fitzgerald, catalog no. 70R-49334), human ALKl (1:100, R&D, catalog no. AF370), mouse ALKl (1:50, R&D, catalog no. AF770), p-SMAD l/5 (1:500, Cell Signaling, catalog no. 9516), human CD31 (1:200, Santa Cruz Biotechnology, catalog no. SC-376764), human CD31 (1:200, Abcam, catalog no. ab28364), mouse CD31 (1:200, BD Biosciences, catalog no. 553370), CD68 (1:200, AbD Serotec, catalog no. MCA1957), SMA (1:200, Santa Cruz Biotechnology, catalog no. SC-53015), VE-Cad (1:200, BD Biosciences, catalog no. 555289), IB4 (Life Technologies, catalog no. 121412), GFP (1:200, lnvitrogen, catalog no. A-21311), LDLR (1:500, Abcam, catalog no. ab30532) and SR-Bl (1:200, Abcam, catalog no. ab137829).

Secondary antibodies were: donkey anti-rat Alexa Fluor-488 (1:500, Invitrogen, catalog no. A21208), donkey anti-rabbit Alexa Fluor-488 (1:500, Invitrogen, catalog no. A21206), donkey anti-goat Alexa Fluor-488 (1:500, Invitrogen, catalog no. A11055), donkey anti-rabbit Alexa Fluor-594 (1:500, Invitrogen, catalog no. A21207), donkey anti-mouse Alexa Fluor-594 (1:500, Invitrogen, catalog no. A21203), donkey anti-rat Alexa Fluor-594 (1:500, Invitrogen, catalog no. A21209), isolectin GS-IB4 Alexa Fluor-488 (1:500, Invitrogen, catalog no. I21411) and isolectin GS-IB4 Alexa Fluor-594 (1:500, Invitrogen, catalog no. I21413).

### Statistical analysis

The animal sample size for each study was chosen based on literature documentation of similar well-characterized experiments. The number of animals used in each study is listed in the figure legends. No inclusion or exclusion criteria were used and studies were blinded to investigators or formally randomized. All data are shown as mean ± s.e.m. and were analyzed using two-tailed, unpaired Student’s *t*-test, one-way analysis of variance (ANOVA) with Sidak’s multiple comparisons test or two-way ANOVA with Tukey’s multiple comparisons test. Data distribution was assumed to be normal but this was not formally tested. Analysis was performed with GraphPad Prism software v.9.

### Reporting summary

Further information on research design is available in the Nature Portfolio Reporting Summary linked to this article.

### Supplementary information

Supplementary Information Supplementary Tables 1 and 2.

Reporting Summary

### Source data

Source Data Fig. 1 Statistical source data.

Source Data Fig. 2 Statistical source data.

Source Data Fig. 3 Statistical source data.

Source Data Fig. 4 Statistical source data.

Source Data Extended Data Fig. 1 Statistical source data.

Source Data Extended Data Fig. 2 Statistical source data.

Source Data Extended Data Fig. 3 Statistical source data.

Source Data Extended Data Fig. 4 Statistical source data.

Source Data Extended Data Fig. 5 Statistical source data.

Source Data Extended Data Fig. 6 Statistical source data.

Source Data Extended Data Fig. 7 Statistical source data.

Source Data Extended Data Fig. 8 Statistical source data.

Source Data Extended Data Fig. 9 Statistical source data.

Source Data Extended Data Fig. 10 Statistical source data.

Source Data Extended Data Fig. 11 Statistical source data.

Source Data Figs. 1 and 2 and Extended Data Figs. 2, 5, 6 and 9 Unprocessed western blots for Figs. 1 and 2 and Extended Data Figs. 2, 5, 6, and 9.

## Data Availability

All sequencing data are available in the GEO (accession nos. GSE43292 for Microarray and GSE159677 for single-cell analysis). All other data used in the present study are included in the main text and associated files. Source data are provided with this paper.
