# Supplementary material for: Genetic or therapeutic neutralization of ALK1 reduces LDL transcytosis and atherosclerosis in mice
Source: Nat Cardiovasc Res. 2023 May 11;2(5):438–48. doi: 10.1038/s44161-023-00266-2 (PMC11358031; doi:10.1038/s44161-023-00266-2)
Supplement: Supplementary file 1 — Supplementary Tables 1 and 2. [file 44161_2023_266_MOESM1_ESM.pdf]

nature cardiovascular research

Letter

https://doi.org/10.1038/s44161-023-00266-2

Genetic or therapeutic neutralization of ALK1 reduces LDL transcytosis and atherosclerosis in mice

In the format provided by the
authors and unedited

Table of Contents

1. Supplementary Table 1. Hemavet analysis from *Alk1*$^{f/f}$*Ldlr*$^{-/-}$ and *Alk1*$^{i\Delta aEC}$*Ldlr*$^{-/-}$ mice………………..…1
2. Supplementary Table 2. Hemavet analysis from *Ldlr*$^{-/-}$ mice injected with IgG or mAb2……………2

Supplementary Table. 1 Hemavet analyses

	ALK1f/f LDLR KO	n	ALK1f/f Bmx LDLR KO	n	P value
WBC	8.74 ± 2.20	4	13.79 ± 4.22	4	0.08 :ns
Neutrophil	2.10 ± 0.43	4	2.52 ± 0.86	4	0.41 :ns
Leukocyte	6.24 ± 2.06	4	10.6 ± 4.61	4	0.13 :ns
Monocyte	0.3 ± 0.04	4	0.44 ± 0.13	4	0.07 :ns
Eosinophil	0.1 ± 0.17	4	0.13 ± 0.06	4	0.69 :ns
Basophil	0.04 ± 0.04	4	0.06 ± 0.02	4	0.23 :ns

Supplementary Table. 1 ALK1 deletion does not impact on circulating immune cell numbers. Table shows average values of circulating immune cells from $Alk1^{f/f}Ldlr^{-/-}$ and $Alk1^{i\Delta aEC}Ldlr^{-/-}$ mice fed a Western diet for 12 weeks. n=4. Values show mean ± S.E.M. *P* values were calculated by two-tailed unpaired t-test

Supplementary Table. 2 Hemavet analyses

	PBS	n	IgG	n	mAb2	n	P value
WBC	6.07 ± 0.59	6	6.15 ± 1.13	8	6.07 ± 1.12	8	0.93 :ns
Neutrophil	1.18 ± 0.25	6	1.26 ± 0.38	8	1.13 ± 0.36	8	0.88 :ns
Leukocyte	4.65 ± 0.81	6	4.65 ± 0.93	8	4.42 ± 0.70	8	0.81 :ns
Monocyte	0.16 ± 0.08	6	0.23 ± 0.17	8	0.25 ± 0.10	8	0.19 :ns
Eosinophil	0.06 ± 0.04	6	0.03 ± 0.03	8	0.07 ± 0.05	8	0.13 :ns
Basophil	0.01 ± 0.01	6	0.01 ± 0.01	8	0.01 ± 0.01	8	0.08 :ns

Supplementary Table. 2 ALK1 deletion does not impact on circulating immune cell numbers. Table shows average values of circulating immune cells from *Ldlr*$^{-/-}$ mice fed a Western diet for 12 weeks with IgG or mAb2 treatment. n=8. Values show mean ± S.E.M. *P* values were calculated by two-tailed unpaired t-test.
