# Supplementary material for: Genetic or therapeutic neutralization of ALK1 reduces LDL transcytosis and atherosclerosis in mice
Source: Nat Cardiovasc Res. 2023 May 11;2(5):438–48. doi: 10.1038/s44161-023-00266-2 (PMC11358031; doi:10.1038/s44161-023-00266-2)
Supplement: Supplementary file 2 — Reporting Summary [file 44161_2023_266_MOESM2_ESM.pdf]

nature portfolio

Corresponding author(s): WC Sessa

Last updated by author(s): March.25.2023

Reporting Summary

Nature Portfolio wishes to improve the reproducibility of the work that we publish. This form provides structure for consistency and transparency in reporting. For further information on Nature Portfolio policies, see our Editorial Policies and the Editorial Policy Checklist.

Statistics

For all statistical analyses, confirm that the following items are present in the figure legend, table legend, main text, or Methods section.

n/a	Confirmed	
☐	☒	The exact sample size (*n*) for each experimental group/condition, given as a discrete number and unit of measurement
☒	☐	A statement on whether measurements were taken from distinct samples or whether the same sample was measured repeatedly
☐	☒	The statistical test(s) used AND whether they are one- or two-sided<br>*Only common tests should be described solely by name; describe more complex techniques in the Methods section.*
☒	☐	A description of all covariates tested
☐	☒	A description of any assumptions or corrections, such as tests of normality and adjustment for multiple comparisons
☐	☒	A full description of the statistical parameters including central tendency (e.g. means) or other basic estimates (e.g. regression coefficient) AND variation (e.g. standard deviation) or associated estimates of uncertainty (e.g. confidence intervals)
☒	☐	For null hypothesis testing, the test statistic (e.g. *F*, *t*, *r*) with confidence intervals, effect sizes, degrees of freedom and *P* value noted<br>*Give P values as exact values whenever suitable.*
☒	☐	For Bayesian analysis, information on the choice of priors and Markov chain Monte Carlo settings
☒	☐	For hierarchical and complex designs, identification of the appropriate level for tests and full reporting of outcomes
☒	☐	Estimates of effect sizes (e.g. Cohen's *d*, Pearson's *r*), indicating how they were calculated

Our web collection on statistics for biologists contains articles on many of the points above.

Software and code

Policy information about availability of computer code

Data collection	Atherosclerosis lesion area images were collected by Leica histology microscope,western blot data by LI-COR (Odyssey Clx platform), and Confocal microscopy data by Leica LAS-X software (SP5).
Data analysis	*All data analyses were performed using Graphpad Prism version 9. Image J/FIJI (version 2.3.0/1.53q). APT pakage (1.20.0) was used to analyze microarray data.*

For manuscripts utilizing custom algorithms or software that are central to the research but not yet described in published literature, software must be made available to editors and reviewers. We strongly encourage code deposition in a community repository (e.g. GitHub). See the Nature Portfolio guidelines for submitting code & software for further information.

Data

Policy information about availability of data

All manuscripts must include a data availability statement. This statement should provide the following information, where applicable:

- Accession codes, unique identifiers, or web links for publicly available datasets
- A description of any restrictions on data availability
- For clinical datasets or third party data, please ensure that the statement adheres to our policy

Gene Expression Omnibus (GEO,https://www.ncbi.nlm.nih.gov/geo/,accession number GSE43292) was used to analyze microarray data. GEO, (http://pubmed.ncbi.nlm.nih.gov/36224302, accesstion number GSE159677) was used to analyze single cell analysis. There are no restrictions on data availability.

Human research participants

Policy information about studies involving human research participants and Sex and Gender in Research.

Reporting on sex and gender	*Sex-specific or gender-specific cardiovascular atherosclerosis progression was not analyzed in this study. No information on sex or gender has been collected but a consent was obtained for specimen collection.*
Population characteristics	*Not applicable because the specimens were derived from a single patient. with a different levels of clinical disease.*
Recruitment	*Not applicable.*
Ethics oversight	Research protocols for consented tissue acquisition were approved by the Institutional Review Boards of Yale University (Protocol ID: 2023-07995).

Note that full information on the approval of the study protocol must also be provided in the manuscript.

Field-specific reporting

Please select the one below that is the best fit for your research. If you are not sure, read the appropriate sections before making your selection.

[X] Life sciences [ ] Behavioural & social sciences [ ] Ecological, evolutionary & environmental sciences

For a reference copy of the document with all sections, see nature.com/documents/nr-reporting-summary-flat.pdf

Life sciences study design

All studies must disclose on these points even when the disclosure is negative.

Sample size	*The number of mice used were 10-12 mice per group and the number of independent mice employed is stated in the figure legend. The sample size were determined by the guideline (Arterioscler Thromb Vase Biol. 2017;37:e131-e157. DOI:10.1161/ATVB.0000000000000062.).*
Data exclusions	*No data were excluded from analysis.*
Replication	*In vitro data were collected from at least 3 independent experiments and in vivo mouse studies were repeated in 2-3 separate cohorts. All experimental findings were successfully repeated by authors.*
Randomization	*Within all genotype groups, age- and sex-matched animals were randomly assigned to the experimental groups.*
Blinding	*The investigators could not be blinded for antibody treatment because each group needs to receive consecutive treatment. However, the investigators were blinded for detailed tissue analysis once data were collected.*

Reporting for specific materials, systems and methods

We require information from authors about some types of materials, experimental systems and methods used in many studies. Here, indicate whether each material, system or method listed is relevant to your study. If you are not sure if a list item applies to your research, read the appropriate section before selecting a response.

Materials & experimental systems

n/a	Involved in the study
☐	☒ Antibodies
☐	☒ Eukaryotic cell lines
☒	☐ Palaeontology and archaeology
☐	☒ Animals and other organisms
☒	☐ Clinical data
☒	☐ Dual use research of concern

Methods

n/a	Involved in the study
☒	☐ ChIP-seq
☐	☐ Flow cytometry
☐	☐ MRI-based neuroimaging

Antibodies

Antibodies used

Anti-ALKI blocking (Genovac, not commercially available), ApoB (1:100, #K23300R, Meridian Life Science, Inc), HSP90 (1:500, #610419, BO Biosciences), human ALKI (1:500, #70R-49334, Fitzgerald), human ALKI (1:100, #AF370, R&D), mouse ALKI (1:50, #AF770, R&D), p-SMADI/5 (1:500, #9516, Cell signaling), human CD31 (1:200, #SC-376764, Santa Cruz Biotechnology), human CD31 (1:200, #ab28364, Abcam), mouse CD31 (1:200, #553370, BO Biosciences), CD68 (1:200, #MCA1957, AbD Serotec), SMA (1:200, #SC-53015, Santa Cruz Biotechnology), VE-CAD (1:200, #555289, BO Biosciences), IB4 (#121412, Life Technologies), GFP (1:200, #A-21311, Invitrogen), LDLR (1:500, #ab30532, Abeam), SR-BI (1:200, #ab137829, Abeam).

Validation

In addition to the validation provided by the commercial sources, we validated the specificity of the antibodies by RNAi deletion of the protein being detected.

Eukaryotic cell lines

Policy information about cell lines and Sex and Gender in Research

Cell line source(s)

Primary human coronary artery endothelial cells (HCAECs) were purchased from PromoCell (cat#: C-12221) and human umbilical vein endothelial cells (HUVECs) were obtained from the Yale University Vascular Biology and Therapeutics Core Facility.

Authentication

Flow cytometry and immunostaining for endothelial cell markers such as CD31 and VE-CAD were performed to authenticate HUVECs.

Mycoplasma contaminatio

The cells were tested negative for mycoplasma.

Commonly misidentified l (See ICLAC register)

No commonly misidentified cell lines were used.

Animals and other research organisms

Policy information about studies involving animals; ARRIVE guidelines recommended for reporting animal research, and Sex and Gender in Research

Laboratory animals

All mouse strains (The ALK1f/f, BmxCreERT, LDLR null, LDLR het) were maintained on a C57BL/6 background. All mouse strains are reported in Method: "Animal models". Only adult male mice (6-7 weeks) were used. Mice used in all experiments were sex and age matched and kept in individually ventilated cages in pathogen-free rooms. Mice were housed in the Yale Animal Facility, fed normal chow (Harlan Teklad, cat#: rodent diet 2018), and were kept at 25 °C on a 12-h light/dark cycle.

Wild animals

Wild animals were not used.

Reporting on sex

Only male mice were used to reduce variability.

Field-collected samples

No field-collected samples were used.

Ethics oversight

Yale Institutional Animal Care & Use Committee (IACUC) approved and guided protocol.

Note that full information on the approval of the study protocol must also be provided in the manuscript.

Dual use research of concern

Policy information about dual use research of concern

Hazards

Could the accidental, deliberate or reckless misuse of agents or technologies generated in the work, or the application of information presented in the manuscript, pose a threat to:

No	Yes	
X	☐	Public health
X	☐	National security
X	☐	Crops and/or livestock
X	☐	Ecosystems
X	☐	Any other significant area

Experiments of concern

Does the work involve any of these experiments of concern:

No	Yes	
X	☐	Demonstrate how to render a vaccine ineffective
X	☐	Confer resistance to therapeutically useful antibiotics or antiviral agents
X	☐	Enhance the virulence of a pathogen or render a nonpathogen virulent
X	☐	Increase transmissibility of a pathogen
X	☐	Alter the host range of a pathogen
X	☐	Enable evasion of diagnostic/detection modalities
X	☐	Enable the weaponization of a biological agent or toxin
X	☐	Any other potentially harmful combination of experiments and agents
