# Supplementary material for: Genetic or therapeutic neutralization of ALK1 reduces LDL transcytosis and atherosclerosis in mice
Source: Nat Cardiovasc Res. 2023 May 11;2(5):438–48. doi: 10.1038/s44161-023-00266-2 (PMC11358031; doi:10.1038/s44161-023-00266-2)

Source Data Fig 1

Source Data: Fig 1g. Uncropped images

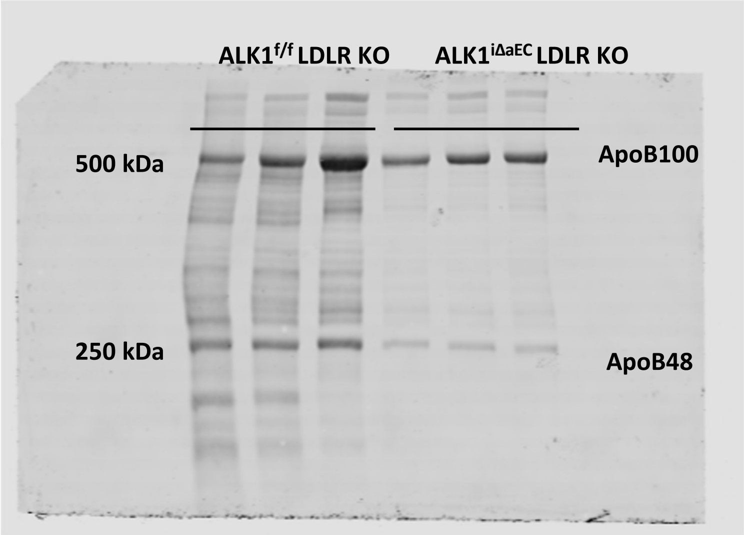

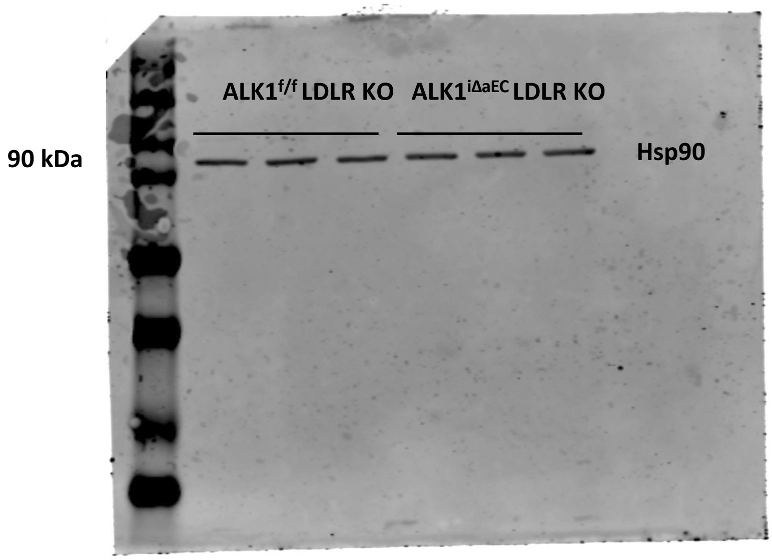

Source Data Fig 2

Source Data: Fig 2a. Uncropped images

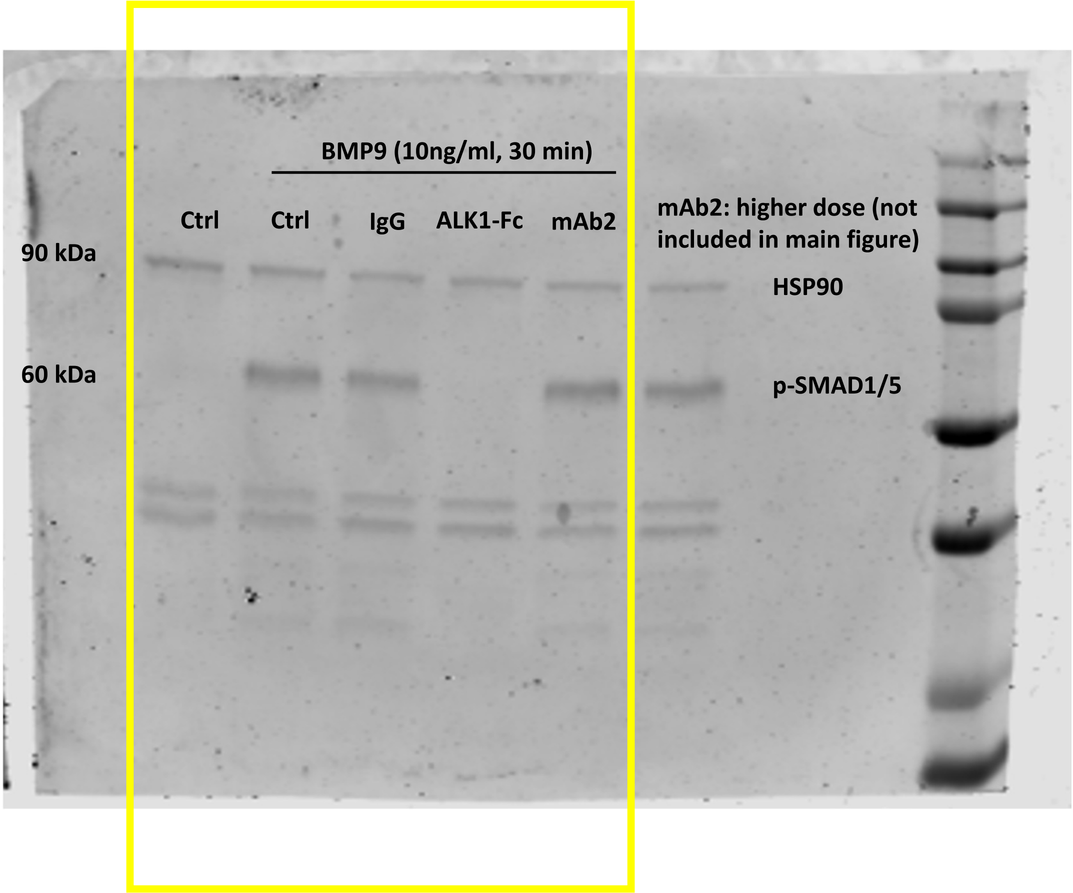

Source Data Extended Data Fig 2

Source Data: Extended Data Fig 2a. Uncropped images

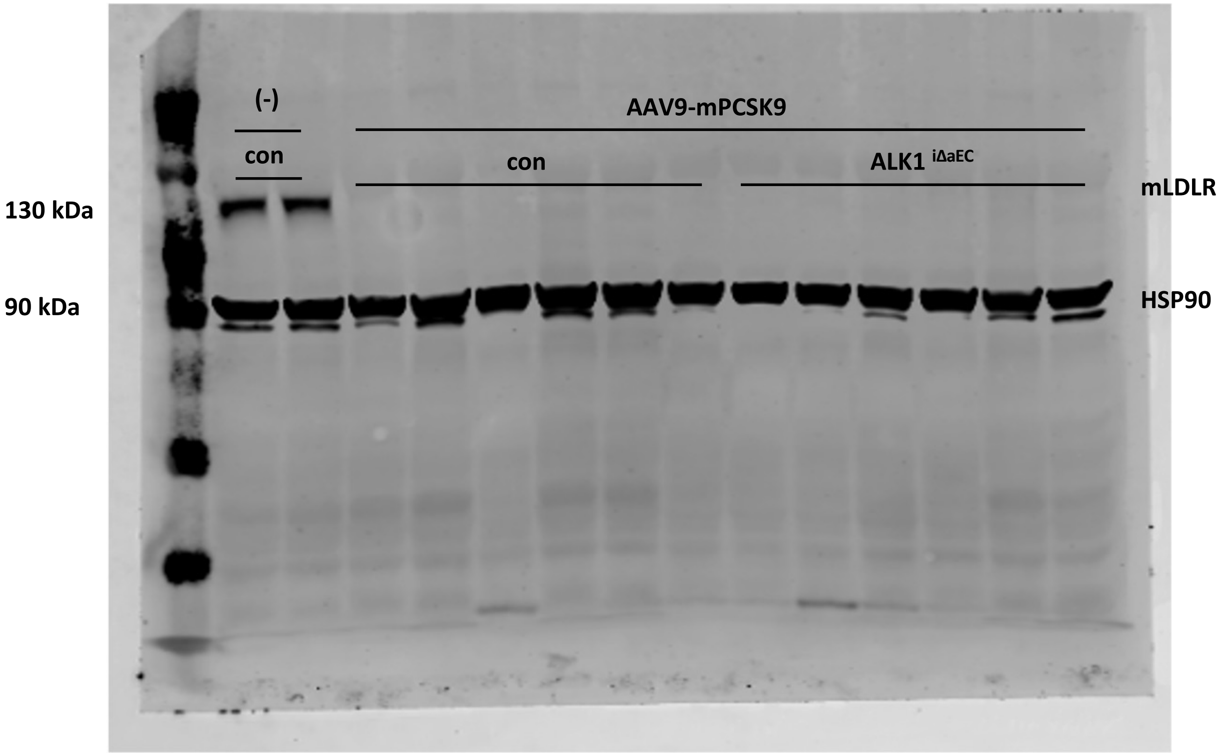

Source Data Extended Data Fig 5

Source Data: Extended Data Fig 5a. Uncropped images

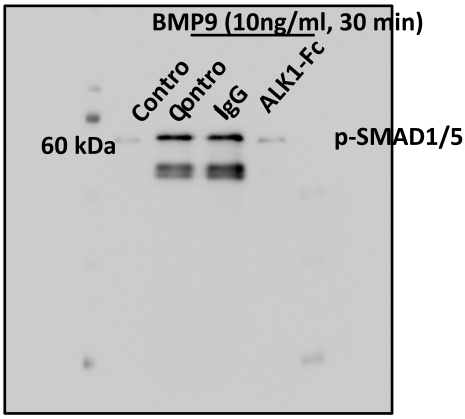

Supernatants of clones from 82 to 89

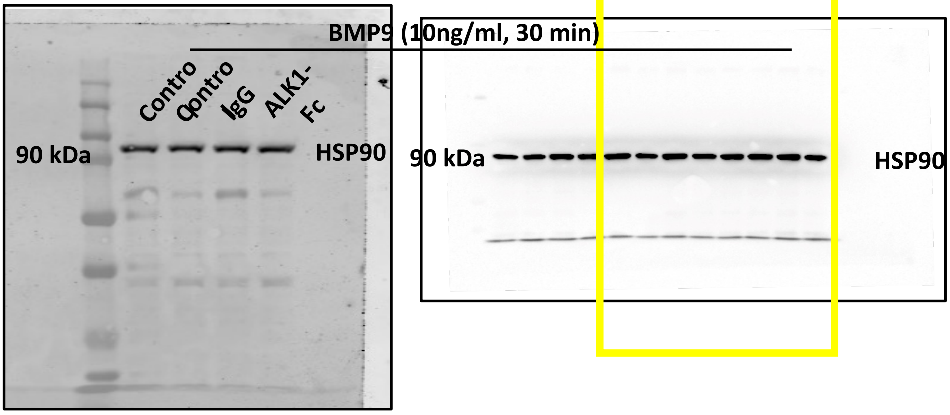

Source Data: Extended Data Fig 5b. Uncropped images

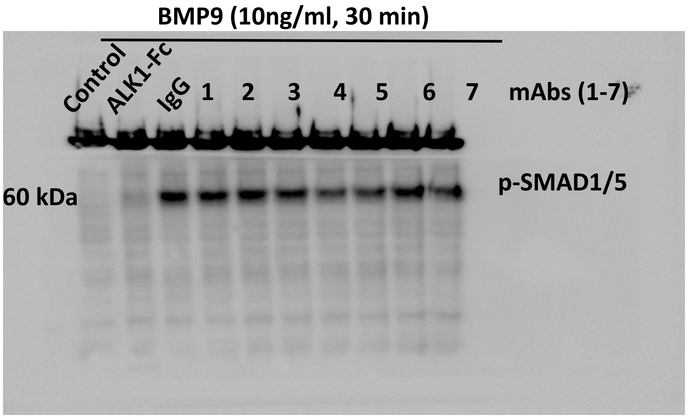

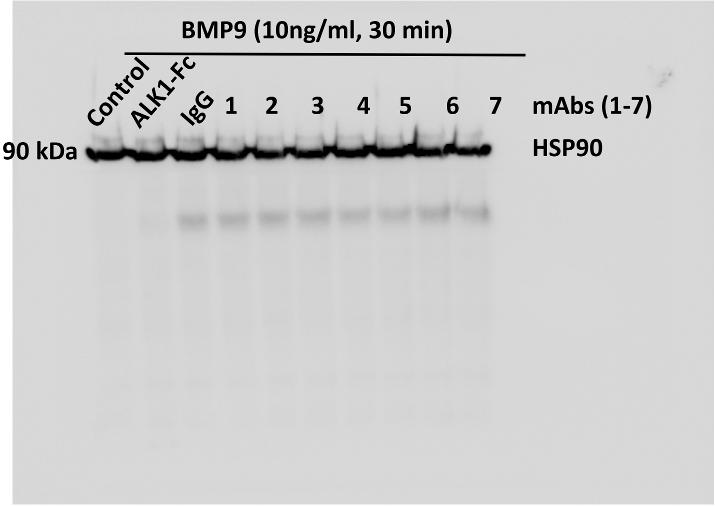

Source Data Extended Data Fig 5

Source Data: Extended Data Fig 5d. Uncropped images

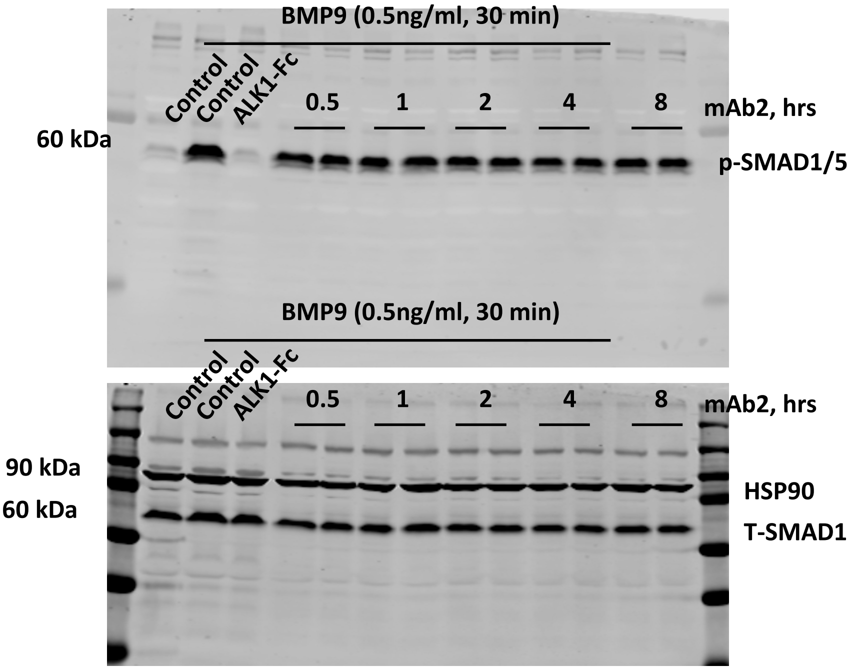

Source Data: Extended Data Fig 5f. Uncropped images

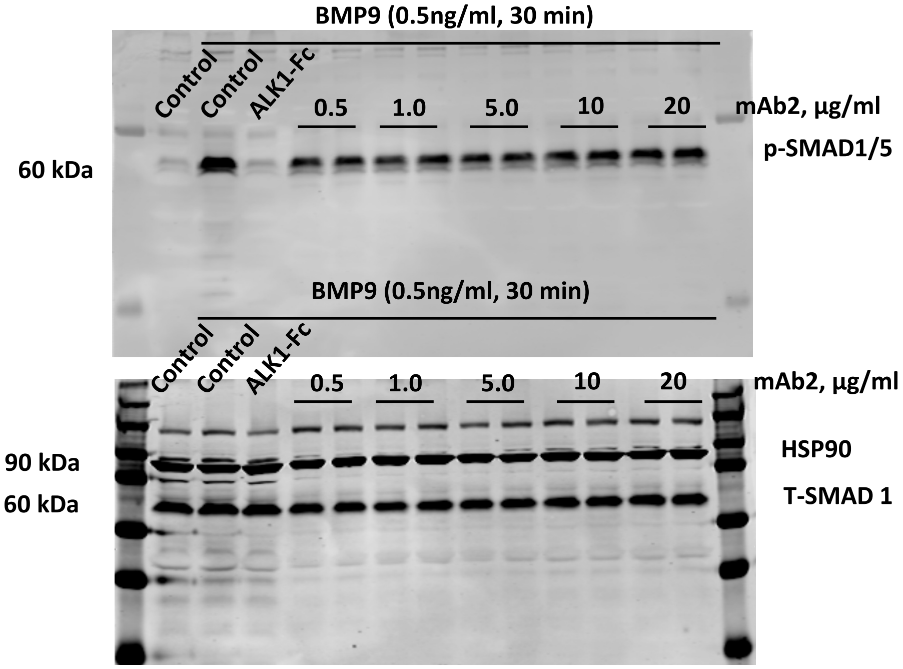

Source Data Extended Data Fig 5

Source Data: Extended Data Fig 5h. Uncropped images

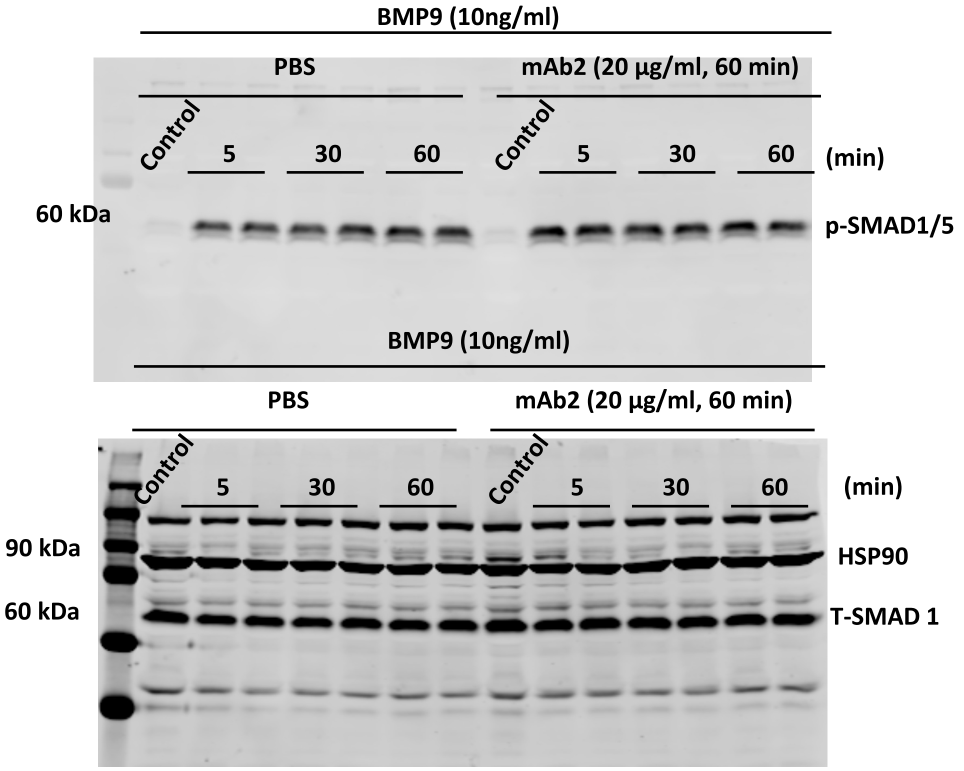

Source Data: Extended Data Fig 5j. Uncropped images

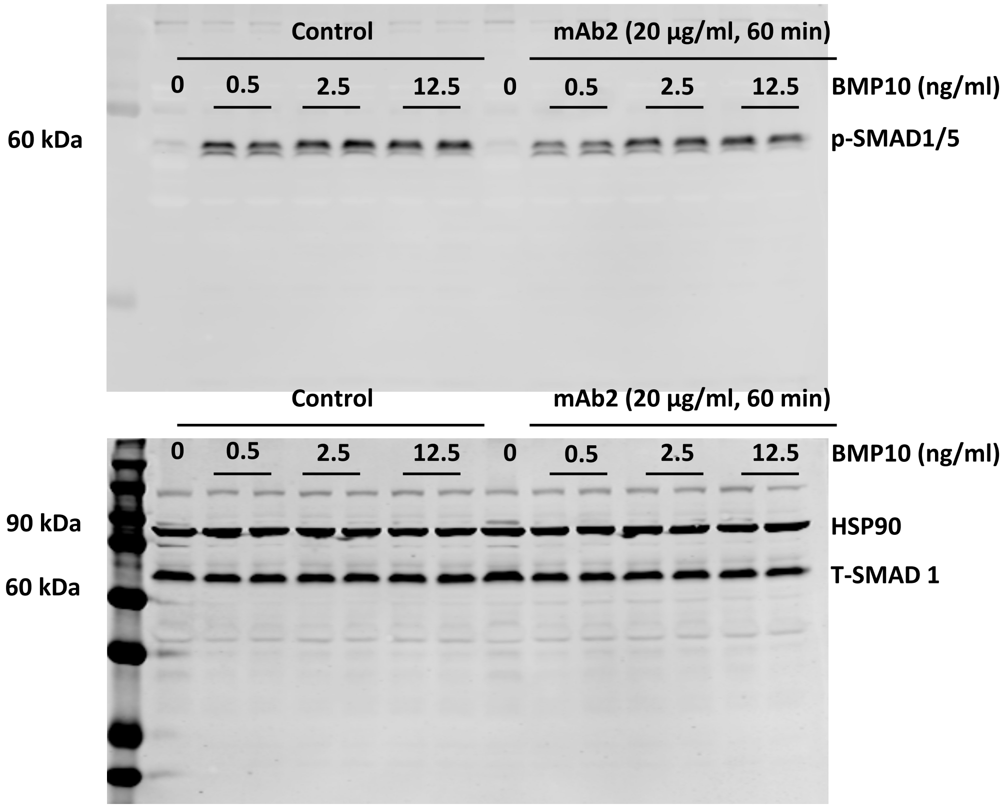

Source Data Extended Data Fig 5

Source Data: Extended Data Fig 5l. Uncropped images

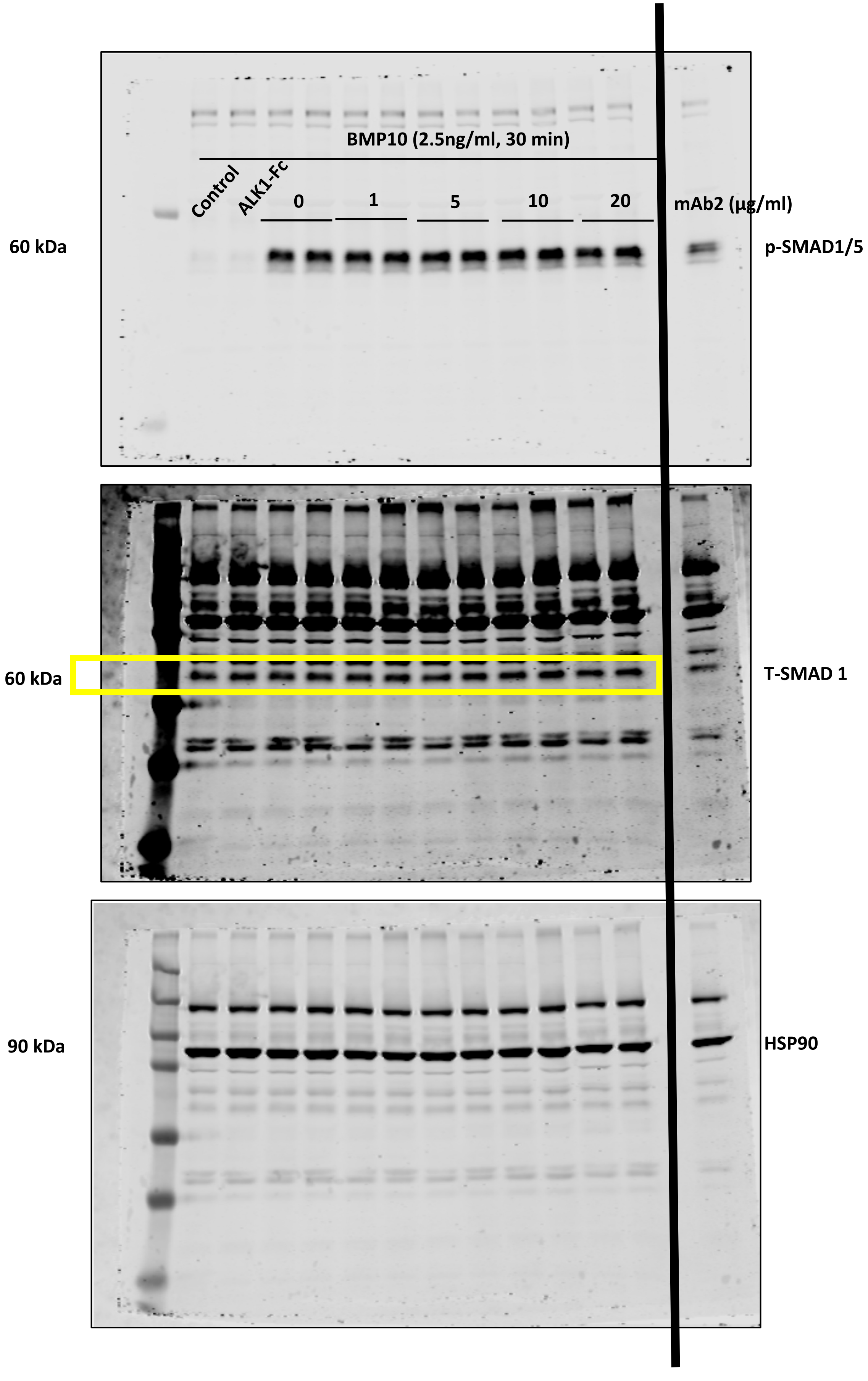

Source Data Extended Data Fig 6

Source Data: Extended Data Fig 6c. Uncropped images

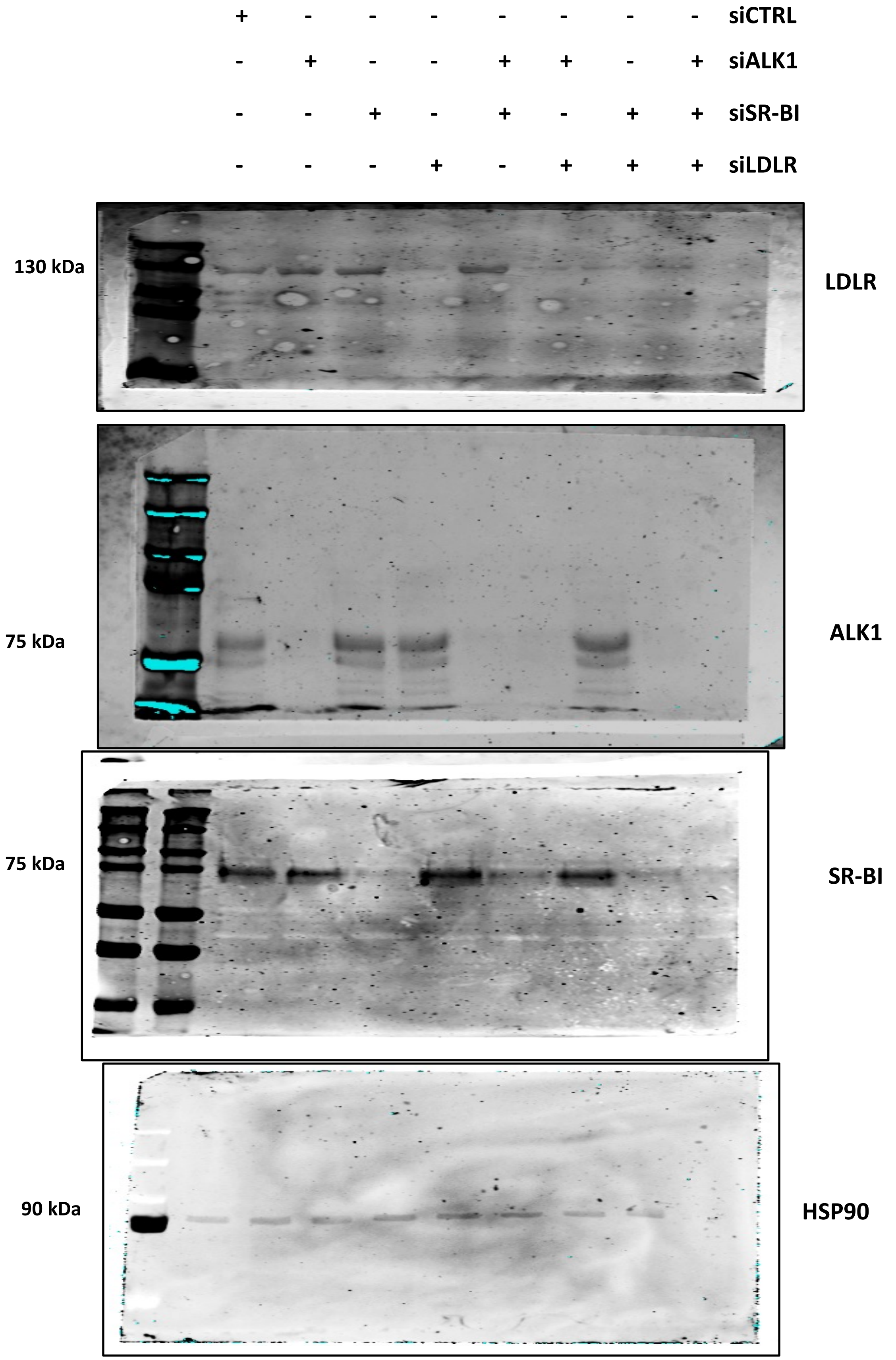

Source Data Extended Data Fig 9

Source Data: Extended Data Fig 9f. Uncropped images

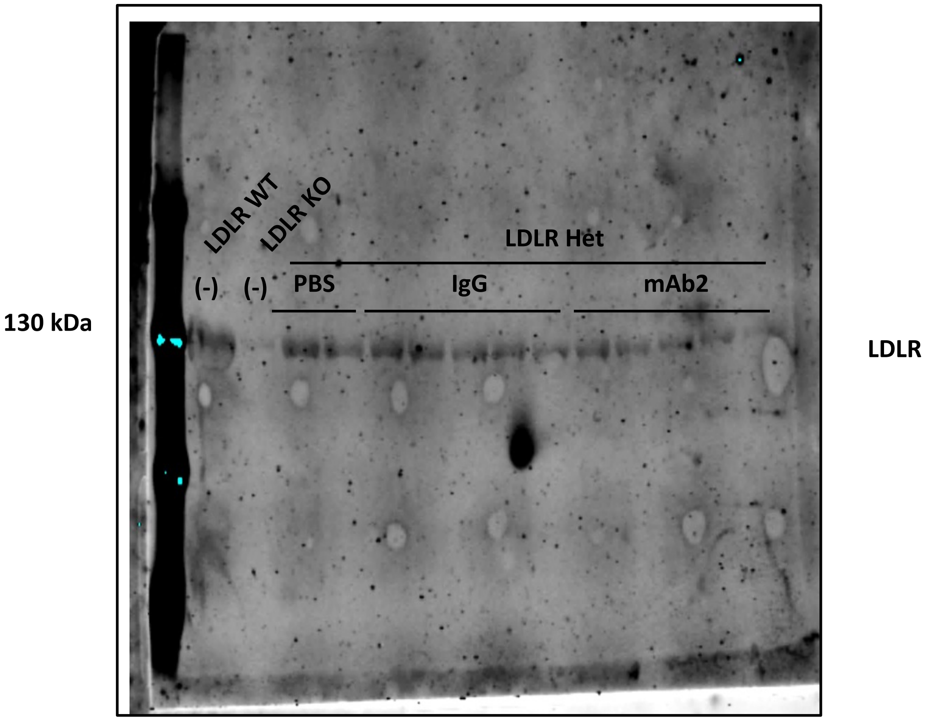

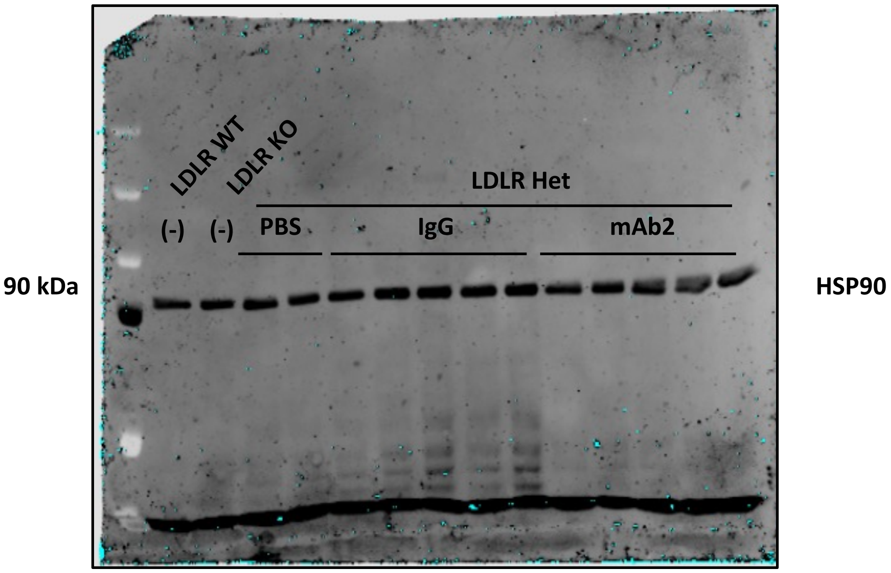

Supplement: Supplementary file 18 — Unprocessed western blots for Figs. 1 and 2 and Extended Data Figs. 2, 5, 6, and 9. [file 44161_2023_266_MOESM18_ESM.pdf]
